# An exploratory identification of biological markers of chronic musculoskeletal pain in the low back, neck, and shoulders

**DOI:** 10.1371/journal.pone.0266999

**Published:** 2022-04-15

**Authors:** Codjo Djignefa Djade, Caroline Diorio, Danielle Laurin, Clermont E. Dionne

**Affiliations:** 1 Department of Social and Preventive Medicine, Université Laval, Québec City (Québec), Canada; 2 Centre de recherche du CHU de Québec - Université Laval, Québec City (Québec), Canada; 3 Centre d’excellence sur le vieillissement de Québec (CEVQ) du Centre de recherche en santé durable VITAM, Québec City (Québec), Canada; 4 Faculty of Pharmacy, Université Laval, Québec City (Québec), Canada; 5 Department of Rehabilitation, Faculty of Medicine, Université Laval, Québec City (Québec), Canada; Institute for Advanced Sustainability Studies, GERMANY

## Abstract

**Objectives:**

This study was an in-depth exploration of unique data from a nationally representative sample of adults living in the United States to identify biomarkers associated with musculoskeletal pain.

**Methods:**

We performed secondary analyses of 2003–2004 NHANES data. After a first screening of 187 markers, analyses of 31 biomarkers were conducted on participants aged ≥20 years identified in all counties using the 2000 Census Bureau data (n = 4,742). To assess the association of each biomarker with each pain outcome (acute, subacute and chronic low back, neck, and shoulder pain), analyses were carried out using multivariable logistic regression with adjustments for sex, age and body mass index. Biomarkers were considered as continuous variables and categorized at the median of their distributions.

**Results:**

Pain at any site for ≥24 hours during the past month was reported by 1,214 participants. Of these, 779 mentioned that the pain had lasted for ≥3 months (“chronic pain”). α-carotene, ascorbic acid, β-carotene, mercury and total protein had a statistically significant, inverse association with ≥2 chronic pain sites. Acrylamide, alkaline phosphatase, cadmium, cotinine, glycidamide, homocysteine, retinol, triglycerides and white blood cell count were positively associated with ≥2 chronic pain sites. Few biological markers were associated with acute and subacute pain.

**Conclusions:**

This study identified some biomarkers that were strongly and consistently associated with musculoskeletal pain. These results raise new hypotheses and could have tremendous implications for advancing knowledge in the field. Research on musculoskeletal pain needs to put more effort on the biological dimension of the biopsychosocial model of pain.

## Introduction

Musculoskeletal pain, particularly at the lower back, is one of the main sources of disability-adjusted life years in several countries [1, 2]. Low back pain is a major cause of work absence worldwide [3] and generates one of the heaviest burdens of disease [4]. Our current understanding of musculoskeletal pain is based on the biopsychosocial model of pain that has led to much progress in the past 30 years [5]. However, while a lot of research interest has been directed toward psychosocial determinants, it seems that the “bio” part of the model has been mistreated, as the studies that have been conducted on these specific potential determinants targeted mostly mechanical and clinical variables.

In recent years, research on musculoskeletal pain has shown that work exposures are explaining only a limited fraction of the problem, that children, teenagers and adults, and not only workers in their fifties, suffer from musculoskeletal pain, and that most interventions are useless and do not alter the natural history of the disease [6]. In fact, we have made much progress on excluding potential determinants and interventions, but much less advances on identifying actual ones. Although designing new interventions against musculoskeletal pain and conducting randomized controlled trials on such interventions are useful, the design of efficient interventions would have much better chances if based on a specific understanding of the disease. Musculoskeletal epidemiology still has to identify major determinants of musculoskeletal pain beyond socio-economic deprivation, cigarette smoking, psychological distress, obesity and some specific job exposures (e.g. manual material handling and job’s physical and psychological demands).

A biomarker is a characteristic that can be objectively measured and evaluated as an indicator of normal biological processes, pathogenic processes or pharmacological responses to a therapeutic intervention [7]. In other applications of epidemiology, some biomarkers have been identified that have major clinical and research utility. Because the onerous costs of measuring biomarkers coupled with the numerous candidates considerably limit the opportunity of research, it is possible that we have not yet identified all biological determinants of musculoskeletal pain. Biomarkers of inflammation, vitamins, and pesticides, for example, could lead us to a better understanding of the pathogenic pathways to musculoskeletal pain. We thus conducted this study as a stringent exploration of biomarkers of musculoskeletal pain using very unique data from the National Health and Nutrition Examination Surveys (NHANES). These data document a wide range of biomarkers on a large representative sample of the US population, and as such offer a rare opportunity to study multiple biological variables with high statistical power.

## Materials and methods

### Source of data

Conducted by the National Center for Health Statistics (NCHS) of the Centers for Disease Control and Prevention (CDC), NHANES is a periodic cross-sectional program designed to assess the health and nutritional status of the population of the United States. The sample is representative of the resident civilian non-institutionalized U.S. population [8]. We used NHANES 2003–2004 survey data because it still is the most recent examination that included questions related to pain.

### Data collection

NHANES data collection took place throughout the year and included a household interview and an examination conducted in a Mobile Examination Center (MEC) [9] which occurred within two weeks after the interview. The examination consisted of several questionnaires, and included physical measurements such as blood pressure, dental examination, and collection of blood, hair and urine specimens for laboratory testing [8].

### Identification and recruitment of participants

**Fig 1:**
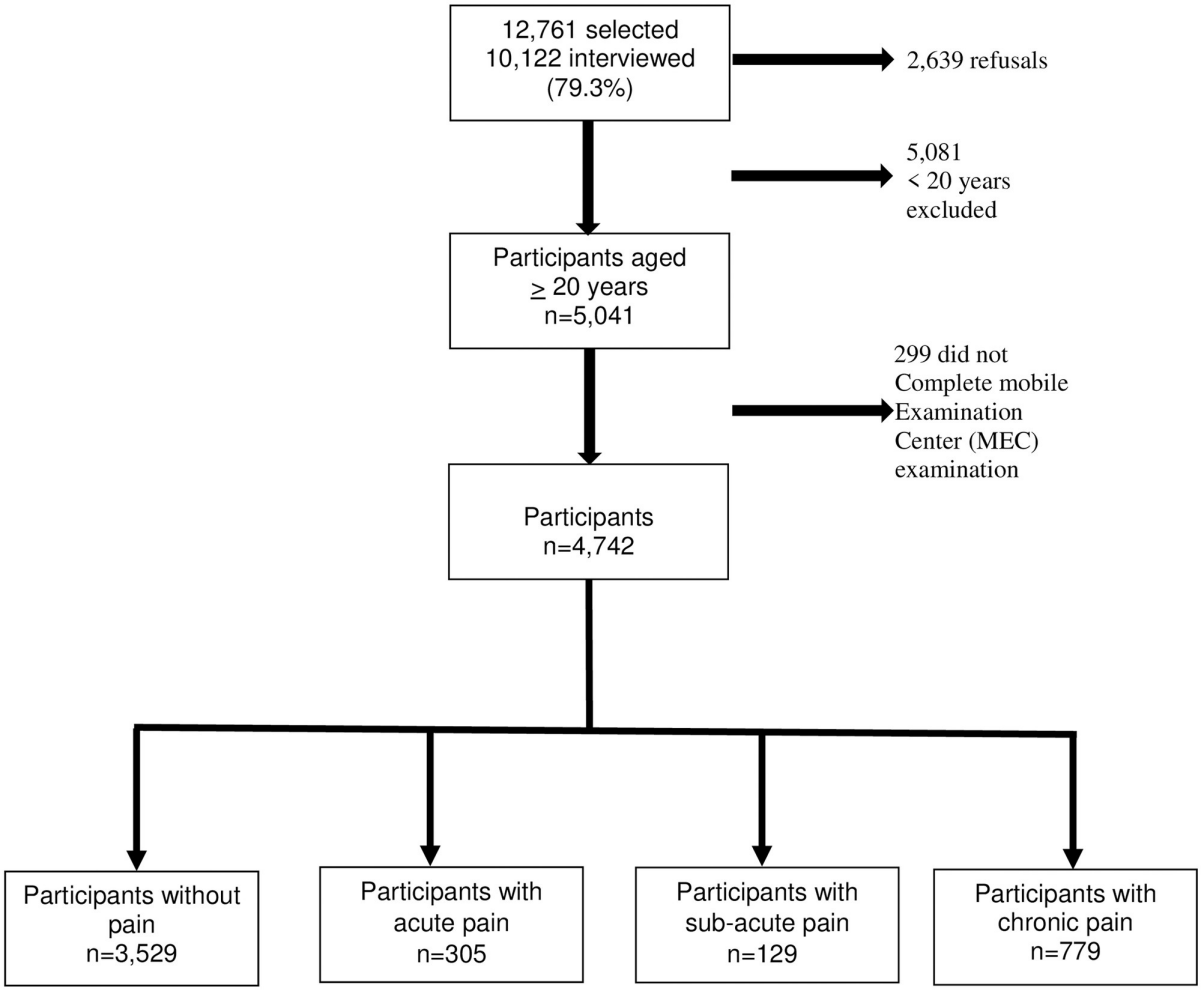
Selection of study participants. N.B: The number of participants with pain includes all pain sites included in NHANES 2003–2004. The question on pain includes all sites of musculoskeletal pain (shoulder, arm, low back, leg, neck, spine, hand, foot…).

NHANES sample is representative of the non-institutionalized civilian population residing in the 50 U.S. states and the District of Columbia [10]. Participants were identified from Primary Sampling Units (PSU) in all counties using the 2000 Census Bureau data. Clusters of households were selected, each person in a selected household was screened for demographic characteristics, and one or more persons per household were chosen for the sample. The analysis was restricted to participants aged ≥20 years (n = 5,041). In this age group, 4,742 (94.1%) answered the questions about miscellaneous pain. Fig 1 shows the details of participation.

### Outcome measures

The outcomes for this study were retrieved from the “Miscellaneous Pain” questionnaire. These dichotomous variables indicated, during the past month, whether or not participants had pain that had lasted for ≥24 hours on a given anatomical site. We selected three anatomical sites: the lower back, neck and shoulder, which are sites most commonly affected by musculoskeletal pain. This definition of pain included acute (duration ≤1 month), subacute (between 1 and 3 months), and “chronic” (i.e. persistent for ≥3 months) episodes [11, 12]. The three sites were chosen because they are the most common; the idea is not to show the mechanism that causes pain, but the fact that a biomarker is associated with several pain sites supports the importance of this biomarker in musculoskeletal pain. Because the physiological processes of a musculoskeletal injury vary by duration, we performed analyses stratified by the duration of pain, with a main focus on chronic pain, since it is responsible for the largest part of the burden of musculoskeletal pain [13].

### Independent variables

#### • Biomarkers

The laboratory components of NHANES 2003–2004 included the collection of various biological and environmental samples. The data collection and reporting systems are integrated within the main NHANES survey database. While the complete blood count analyses were performed in the MEC laboratory, most of the laboratory analyses were conducted off site. Biomarkers documented included bone formation markers, markers of inflammation, metals, vitamins, pesticides, and other environmental markers, among others (see S3 Appendix for complete list). Laboratory procedures used by NHANES were applied according to recognized and valid methods [14]. To avoid over-dispersion, biomarkers with more than 30% missing data were not included in the analyses for the current study.

#### • Other independent variables

The other independent variables considered were taken from the NHANES 2003–2004 demographic variables list. Sex was indicated as female or male. Age was that at the time of the interview. Body mass index (BMI) was calculated from measures taken during the interview, as weight in kilograms divided by height in meters squared and partitioned into four categories:

$$<20;\;20-24.9;\;25-29.9\;\text{and}\;>30\;\text{kg}/{\text{m}}^{2}\;\left(\text{obese}\right)$$

[15, 16].

### Statistical analyses

The general background descriptive characteristics of the participants were computed in terms of frequency and percentage for categorical variables and means (standard deviations—SD) for continuous ones. Biomarkers were characterized by median and interquartile range. To assess the association of each biomarker with each of the three pain outcomes according to the combination of duration and anatomical site, two analyses were carried out. Bivariate and multivariable logistic regressions were performed to produce respectively crude prevalence odds ratios (OR) and adjusted OR (_a_OR) for sex, age and BMI. Due to the numerous biomarkers measured in NHANES, and considering the exploratory nature of the study, we first ran the bivariate analyses using all biomarkers as continuous variables and dichotomized at the median of the distribution and retained only those that were statistically associated with pain on at least two anatomical sites (all durations) for the subsequent analyses. With biomarkers considered continuously, the estimates were computed for the increase of one unit. All estimates were weighted, and variances corrected by considering strata and PSU [17]. Sensitivity analyses were conducted to assess the impacts on the conclusions of using each biomarker categorized into quartiles.

All analyses were conducted using SAS 9.4 *(SAS Institute Inc*., *Cary*, *NC*, *USA*) survey procedures with weight, stratum and cluster provided by NHANES [18]. Statistical significance was fixed at a 0.05 threshold. In agreement with Rothman and Rubin, we did not adjust for multiple comparisons [19, 20]

### Ethics

No ethical approval was required for this study because NHANES data are anonymous and of the public domain.

## Results

Table 1 presents selected characteristics of the 4,742 study participants and the prevalence of pain outcomes. Overall, 52% of participants were females and the mean age was 46.4 years (SD: 0.5). The vast majority was composed of non-Hispanic whites (72.1%), married, or living as married (63.6%) and currently working (60.7%). The proportion of participants who had a high school diploma as the highest level of formal education was 58.3% and 33.3% reported an annual family income of 55,000 USD or more. Almost half (49.2%) of participants were nonsmokers, 21.4% reported daily smoking, 31.7% were obese and 17.7% had more than one comorbidity. Over 25% (n = 1,214) of participants reported pain in at least one musculoskeletal site that had lasted ≥24 hours during the past month: 36.6% at the lower back, 26.4% at the neck, and 24.5% at the shoulder. Among them, 779 (64.2%) mentioned that the pain had lasted ≥3 months (“chronic pain”) [11].

**Table 1:** Selected characteristics of the study sample (n = 4,742).

Variables		N (%)^a^
Sex	Female	2467 (52.0)
	Male	2275 (48.0)
Age	Years, mean (±SD)	46.4 (±0.5)
	20–34	1303 (29.2)
	35–49	1112 (31.3)
	50–64	955 (22.3)
	65–79	923 (12.9)
	80 +	449 (4.2)
Formal education	Less than high school	1402 (18.4)
	High school diploma	2470 (58.3)
	College graduate and above	861 (23.2)
	Missing	9 (0.1)
Race	Mexican American	951 (7.8)
	Other Hispanic	143 (3.6)
	Non-Hispanic white	2510 (72.1)
	Non-Hispanic black	934 (11.2)
	Others	204 (5.4)
Marital status	Never married	795 (17.5)
	Married/Living with partner	2847 (63.6)
	Widowed/Divorced/Separated	1097 (18.8)
	Missing	3 (0.1)
Annual family income (USD)	< 20,000	1535 (23.0)
	20,000–54,999	1865 (39.2)
	≥ 55,000	1139 (33.3)
	Missing	203 (4.5)
Occupational status	Working at a job/business	2344 (60.7)
	Having a job/business but not at work (including layoff)	182 (4.7)
	Looking for work	109 (2.4)
	Not working	2106 (32.3)
	Missing	1 (0.0)
Number of comorbidities	0	2480 (57.6)
	1	1208 (24.7)
	>1	1054 (17.7)
Tobacco use (self-reported)^b^	Non-smoker	2380 (49.2)
	Former smoker	1286 (25.2)
	Occasional smoker	188 (4.1)
	Current daily smoker	882 (21.4)
	Missing	6 (0.1)
Body mass index (BMI)	Kg/m^2^, mean (±SD)	28.2 (±0.15)
	< 20	212 (5.0)
	20–24.9	1266 (28.2)
	25–29.9	1631 (33.5)
	≥ 30	1538 (31.7)
	Missing	95 (1.6)
Pain for more than 24 hours during the past month^c^	Yes	1214 (28.3)
	No	3522 (71.6)
	Don’t know	5 (0.1)
	Missing	1 (0.0)
For how long participant has experienced this pain^d^	Less than a month	305 (27.0)
	At least 1 month but less than 3 months	129 (10.0)
	At least 3 months but less than 1 year	172 (13.7)
	Greater than 1 year	607 (49.3)
	Missing^e^	1 (0.0)
Anatomical sites affected by pain problems^f^ (n = 1,213^g^/4,742)	Neck	308 (26.4)
	Shoulder	288 (24.5)
	Lower back	451 (36.6)
	Other sites	497 (40.6)

^a^ n are actual frequencies, while % are weighted to take the sampling design into account. The sum of percentages may exceed 100% because of rounding.

^b^ Definitions of categories based on Copley et al.: Copley C, O’Connor S, on behalf of the National Advisory Group on Monitoring and Evaluation. Canadian Tobacco Control Research Initiative. Indicators for monitoring tobacco control: a resource for decision-makers, evaluators and researchers. Toronto: Canadian Tobacco Control Research Initiative 2006.

^c^ Musculoskeletal pain at all specified sites in NHANES (shoulder, arm, low back, leg, neck, spine, hand, foot…).

^d^ The sample for this variable includes participants who mentioned having had a pain problem that lasted ≥24 hours in the past month (N = 1,214).

^e^ One participant declared having had a problem with pain that lasted ≥24 hours in the past month but not in the three sites of interest. He/she has been considered missing.

^f^ The categories are not mutually exclusive and this number includes all pain sites considered in NHANES.

^g^ Among the 1,214 participants who responded they had pain that lasted ≥24 hours in the past month, there was one who did not specify the site of pain; therefore, these percentages were calculated on 1,213 subjects.

Table 2 presents the median and interquartile range of the biomarkers retained following the preliminary analyses (i.e. all the biomarkers that were statistically associated with at least two pain sites—all durations—when considered as continuous variables or dichotomized at the median of the distribution in bivariate logistic regression). These biomarkers are subdivided into groups of 1) vitamins and urinary markers, 2) cadmium, lead and total mercury, 3) blood count, marker of inflammation, cotinine and homocysteine, and 4) standard biochemicals. Biomarkers belonging to the groups of brominated flame retardants, metals in urine, organophosphorus insecticides, pesticides environmental in urine, pesticides—organochlorine metabolites, phthalates, phytoestrogens and polyfluoroalkyl chemicals were not statistically associated with any two of the three musculoskeletal pain sites considered. Because crude and adjusted models provided similar results, only those from adjusted models are presented.

**Table 2:** Specific biomarkers considered in main analyses (n = 4,742).

Variables	N (%)^a^	
**Vitamins and urinary markers** ^b^		
Acrylamide (pmoL/G Hb)	N for value above median	1949 (47.6)
	Median (Q1 –Q3)	53.4 (41.2–84.6)
	Missing	649 (13.7)
Glycidamide (pmoL/G Hb)	N for value above median	1979 (52.3)
	Median (Q1 –Q3)	57.8 (40.5–84.8)
	Missing	590 (12.4)
Albumin, urine (ug/mL)	N for value above median	2463 (55.8)
	Median (Q1 –Q3)	6.84 (3.54–13.48)
	Missing	147 (3.0)
Ascorbic acid (umol/L) (Vitamin C)	N for value above median	2252 (50.7)
	Median (Q1 –Q3)	54.4 (31.7–70.7)
	Missing	304 (6.4)
Retinol (ug/dL) (Vitamin A)	N for value above median	2052 (46.1)
	Median (Q1 –Q3)	58.5 (48.5–69.5)
	Missing	289 (6.1)
α-Tocopherol (μg/dL) (Vitamin E)	N for value above median	2265 (50.9)
	Median (Q1 –Q3)	1205.21 (957.02–1616.51)
	Missing	289 (6.1)
α-Carotene (μg/dL)	N for value above median	2300 (51.7)
	Median (Q1 –Q3)	2.5 (1.3–4.8)
	Missing	289 (6.1)
β-Carotene (μg/dL)	N for value above median	2334 (52.5)
	Median (Q1 –Q3)	11.9 (7.0–21.5)
	Missing	296 (6.2)
**Cadmium, lead and total mercury (blood test)**		
Cadmium (μg/L)	N for value above median	2452 (54.2)
	Median (Q1 –Q3)	0.3 (0.2–0.6)
	Missing	217 (4.6)
Lead (μg/dL)	N for value above median	2591 (57.3)
	Median (Q1 –Q3)	1.4 (1.0–2.2)
	Missing	217 (4.6)
Mercury, total (μg/L)	N for value above median	2216 (49.0)
	Median (Q1 –Q3)	0.9 (0.4–1.9)
	Missing	217 (4.6)
**Blood count, marker of inflammation, cotinine and homocysteine**		
White blood cell count (1000 cells/μL)	N for value above median	2298 (50.7)
	Median (Q1 –Q3)	7.0 (5.7–8.4)
	Missing	212 (4.5)
Platelet count SI (1000 cells/μL)	N for value above median	2153 (47.5)
	Median (Q1 –Q3)	258.5 (220.7–302.4)
	Missing	212 (4.5)
C-reactive protein (mg/dL)	N for value above median	2455 (54.7)
	Median (Q1 –Q3)	0.2 (0.1–0.4)
	Missing	255 (5.4)
Cotinine (ng/mL)	N for value above median	2084 (46.6)
	Median (Q1 –Q3)	0.1 (0.0–64.9)
	Missing	266 (5.6)
Homocysteine (μmol/L)	N for value above median	2385 (52.9)
	Median (Q1 –Q3)	8.3 (6.9–10.3)
	Missing	233 (4.9)
**Standard biochemicals**		
Gamma glutamyl transferase (U/L)	N for value above median	2410 (54.1)
	Median (Q1 –Q3)	18.8 (13.1–29.4)
	Missing	288 (6.1)
Alkaline phosphatase (U/L)	N for value above median	2478 (55.6)
	Median (Q1 –Q3)	64.49 (53.21–78.72)
	Missing	287 (6.1)
Total calcium (mg/dL)	N for value above median	2571 (57.7)
	Median (Q1 –Q3)	9.50 (9.27–9.72)
	Missing	287 (6.1)
Direct HDL-cholesterol (mg/dL)	N for value above median	2308 (51.6)
	Median (Q1 –Q3)	51.1 (41.8–62.7)
	Missing	267 (5.6)
Total cholesterol (mg/dL)	N for value above median	2300 (51.4)
	Median (Q1 –Q3)	197.7 (172.1–226.3)
	Missing	266 (5.6)
Lactate dehydrogenase LDH (U/L)	N for value above median	2388 (53.7)
	Median (Q1 –Q3)	123.2 (110.0–139.4)
	Missing	292 (6.2)
Phosphorus (mg/dL)	N for value above median	2321 (52.1)
	Median (Q1 –Q3)	3.7 (3.4–4.1)
	Missing	288 (6.1)
Total protein (g/L)	N for value above median	2375 (50.1)
	Median (Q1 –Q3)	71.2 (71.2–74.3)
	Missing	290 (6.1)
Triglycerides (mg/dL)	N for value above median	2330 (50.1)
	Median (Q1 –Q3)	107.2 (71.2–164.8)
	Missing	290 (6.1)
Uric acid (mg/dL)	N for value above median	2258 (47.6)
	Median (Q1 –Q3)	5.2 (4.3–6.2)
	Missing	289 (6.1)
Sodium (mmol/L)	N for value above median	2784 (62.5)
	Median (Q1 –Q3)	138.7 (137.4–140.0)
	Missing	288 (6.1)
Potassium (mmol/L)	N for value above median	2414 (54.2)
	Median (Q1 –Q3)	3.9 (3.7–5.9)
	Missing	288 (6.1)
Chloride (mmol/L)	N for value above median	2448 (54.9)
	Median (Q1 –Q3)	103.3 (101.6–104.8)
	Missing	287 (6.1)
Globulin (g/dL)	N for value above median	2708 (60.8)
	Median (Q1 –Q3)	2.8 (2.6–3.1)
	Missing	290 (6.1)
Bicarbonate (mmol/L)	N for value above median	2538 (57.0)
	Median (Q1 –Q3)	24.4 (22.7–25.6)
	Missing	287 (6.1)

^a^ As the statistics have been weighted as recommended by NHANES, one can note a little unbalancing of proportions in the classes.

^b^ The medians have been calculated with the *proc surveymeans* of SAS weighted. While the percentages of data above the median were calculated on existing data, the percentages of missing data were calculated on total n (4,742).

Table 3 presents _a_ORs for each biomarker retained in the first analysis step (continuous or dichotomized distribution) with the three chronic pain sites (n = 4,307). Only those remaining associated with at least two chronic pain sites after the second analysis step (dichotomized at the median or continuously) are described below.

- Vitamins and urinary markers
  In this group of biomarkers, only six were associated with two (α-carotene, β-carotene, and ascorbic acid (vitamin C)) or three (acrylamide, glycidamide and retinol (vitamin A)) chronic pain sites. Among these, inverse associations were observed for median levels: α-carotene with chronic low back pain (_a_OR: 0.6; 95%CI: 0.4–0.8) and chronic shoulder pain (_a_OR: 0.4; 95%CI: 0.3–0.6); β-carotene with chronic low back pain (_a_OR: 0.5; 95%CI: 0.4–0.8) and chronic shoulder pain (_a_OR: 0.6; 95%CI: 0.4–1.0); and ascorbic acid with chronic neck pain (_a_OR: 0.7; 95%CI: 0.6–0.9) and chronic shoulder pain (_a_OR: 0.6; 95%CI: 0.4–0.9). Median levels of albumin and α-tocopherol (vitamin E) were positively associated with only one site of chronic pain.
- Cadmium, lead and total mercury
  Two of these biomarkers were associated with the outcomes: positive associations were observed between cadmium levels and chronic low back pain (_a_OR: 1.5; 95%CI: 1.2–1.8), chronic shoulder pain (_a_OR: 1.5; 95%CI: 1.2–1.8), and chronic neck pain (_a_OR: 1.4; 95%CI: 1.1–1.7), and inverse associations were observed between median levels of total mercury and chronic low back pain (_a_OR: 0.6; 95%CI: 0.5–0.9), chronic neck pain (_a_OR: 0.7; 95%CI: 0.6–0.9) and chronic shoulder pain (_a_OR: 0.6; 95%CI: 0.4–0.9). Levels of lead were associated with only one chronic pain site.
- Blood count, marker of inflammation, cotinine and homocysteine
  When treated as continuous variables, levels of cotinine and white blood cell count were positively associated with all three sites of chronic pain. Homocysteine levels were positively associated with two chronic pain sites, while median levels of platelet count, and C-reactive protein were associated with only one chronic pain site.
- Standard biochemicals
  Biomarkers of standard biochemistry profiles such as phosphorus, sodium, total calcium, and uric acid were not statistically associated with any pain site. Bicarbonate, chloride, lactate dehydrogenase, potassium, cholesterol (direct-HDL and total), gamma glutamyl transferase and globulin were statistically associated with only one site, whereas alkaline phosphatase and total protein levels were associated with two sites. Median levels of triglycerides were positively associated with low back pain (_a_OR: 1.7; 95%CI: 1.2–2.5) and neck pain (_a_OR: 1.6; 95%CI: 1.1–2.4).

**Table 3:** Results of multivariable analyses of the associations between biomarkers retained and the three chronic pain^a^ sites. studied (n = 4,307).

Biomarkers^b^	Class (n)^c^	Low back pain			Shoulder pain			Neck pain		
		Frequency by class (%)	Odds Ratio (95% CI)	*P value*	Frequency by class (%)	Odds Ratio (95% CI)	*P value*	Frequency by class (%)	Odds Ratio (95% CI)	*P value*
Acrylamide (pmoL/G Hb)	3710		1.0035	*0*.0008*		1.0047	*0*.0005*		1.0031	*0*.0153*
			(1.0017–1.0053)			(1.0025–1.0069)			(1.0007–1.0055)	
	0 (1960)	145 (7.4)	1.2 (0.9–1.6)	*0*.*2821*	84 (4.3)	1.8 (1.1–2.9)	*0*.0226*	91 (4.6)	1.5 (1.0–2.3)	*0*.0453*
	1 (1750)	147 (8.4)			109 (6.2)			114 (6.5)		
Glycidamide (pmoL/G Hb)	3763		1.0032	*0*.0128*		1.0054	*0*.0001*		1.0032	*0*.0426*
			(1.0008–1.0057)			(1.0031–1.007)			(1.0001–1.0064)	
	0 (1994)	139 (7.0)	1.4 (1.0–1.8)	*0*.0274*	87 (4.4)	1.7 (1.1–2.6)	*0*.0183*	96 (4.8)	1.4 (0.9–2.1)	*0*.*1250*
	1 (1769)	151 (8.5)			104 (5.9)			110 (6.2)		
Albumin, urine (μg/mL)	4166		1.0003	*0*.0383*		1.0003	*0*.*3124*		1.0001	*0*.*6506*
			(1.000–1.0006)			(0.9997–1.0008)			(0.9996–1.0006)	
	0 (1850)	136 (7.4)	1.1 (0.9–1.5)	*0*.*3192*	91 (4.9)	1.4 (1.0–1.8)	*0*.0375*	103 (5.6)	1.2 (0.9–1.6)	*0*.*1910*
	1 (2316)	185 (8.0)			122 (5.3)			131 (5.7)		
Ascorbic acid (μmol/L) (Vitamin C)	4023		0.9965	*0*.*1430*		0.9938	*0*.0018*		0.9968	*0*.*1979*
			(0.9916–1.0013)			(0.9903–0.9973)			(0.9917–1.0019)	
	0 (1961)	179 (9.1)	0.8 (0.5–1.0)	*0*.*0565*	125 (6.4)	0.6 (0.4–0.9)	*0*.0281*	118 (6.0)	0.7 (0.6–0.9)	*0*.0077*
	1 (2062)	131 (6.4)			84 (4.1)			105 (5.1)		
Retinol (μg/dL) (Vitamin A)	4035		1.0131	*0*.0046*		1.0148	*0*.0091*		1.0189	*0*.0033*
			(1.0047–1.0217)			(1.0042–1.0254)			(1.0073–1.0305)	
	0 (2172)	142 (6.5)	1.5 (1.1–2.1)	*0*.0213*	97 (4.5)	1.4 (1.1–1.8)	*0*.0134*	104 (4.5)	1.7 (1.2–2.3)	*0*.0027*
	1 (1863)	170 (9.1)			113 (6.1)			120 (3.0)		
α-Tocopherol (μg/dL) (Vitamin E)	4035		1.000	*0*.*9787*		0.9999	*0*.*3992*		1.0001	*0*.*5320*
			(0.9998–1.0003)			(0.9996–1.0002)			(0.9998–1.0004)	
	0 (1958)	138 (7.1)	1.1 (0.8–1.4)	*0*.*5648*	84 (4.3)	1.1 (0.7–1.7)	*0*.*6456*	89 (4.6)	1.4 (1.0–2.0)	*0*.0465*
	1 (2077)	174 (8.4)			126 (6.1)			135 (6.5)		
a-Carotene (μg/dL)	4053		0.9495	*0*.*1568*		0.9632	*0*.*3483*		0.9731	*0*.*2929*
			(0.8816–1.0225)			(0.8870–1.0460)			(0.9226–1.0264)	
	0 (1926)	179 (9.3)	0.6 (0.4–0.8)	*0*.0025*	125 (6.5)	0.4 (0.3–0.6)	*<0*.001*	119 (6.2)	0.7 (0.5–1.0)	*0*.*0643*
	1 (2109)	133 (6.3)			85 (4.0)			105 (5.0)		
β-Carotene (μg/dL)	4035		0.9878	*0*.0483*		0.9827	*0*.0459*		0.9909	*0*.*1166*
			(0.9759–0.9999)			(0.9660–0.9996)			(0.9793–1.0026)	
	0 (1891)	177 (9.4)	0.5 (0.4–0.8)	*0*.0012*	116 (6.1)	0.6 (0.4–1.0)	*0*.0375*	114 (6.0)	0.8 (0.5–1.2)	*0*.*2337*
	1 (2144)	135 (6.3)			94 (4.4)			110 (5.1)		
Cadmium (μg/L)	4309		1.4833	*0*.0002*		1.4959	*0*.0003*		1.3561	*0*.0160*
			(1.2457–1.7662)			(1.2454–1.7967)			(1.0675–1.7228)	
	0 (1860)	106 (5.7)	1.7 (1.2–2.3)	*0*.0038*	68 (3.4)	1.9 (1.1–3.2)	*0*.0183*	81 (4.4)	1.4 (0.8–2.5)	*0*.*2335*
	1 (2449)	215 (9.6)			147 (6.6)			149 (6.7)		
Lead (μg/dL)	4102		1.0222	*0*.*4615*		1.0592	*0*.*1300*		1.0971	*0*.0161*
			(0.9609–1.0874)			(0.9812–1.1435)			(1.0200–1.1801)	
	0 (1734)	130 (7.5)	0.9 (0.7–1.2)	*0*.*5168*	78 (4.5)	1.3 (1.0–1.6)	*0*.0386*	99 (5.7)	1.0 (0.7–1.5)	*0*.*9317*
	1 (2368)	191 (8.1)			137 (63.7)			131 (5.5)		
Mercury, total (μg/L)	4102		0.9204	*0*.*4289*		0.8720	*0*.*0884*		0.9253	*0*.*4019*
			(0.7406–1.1439)			(0.7429–1.0235)			(0.7637–1.1210)	
	0 (2094)	192 (9.2)	0.6 (0.5–0.9)	*0*.0074*	123 (5.9)	0.6 (0.4–0.9)	*0*.0271*	125 (6.0)	0.7 (0.6–0.9)	*0*.0073*
	1 (2008)	129 (6.4)			92 (4.6)			105 (5.2)		
Triglycerides (mg/dL)	4036		1.0008	*0*.*0685*		1.0008	*0*.*1556*		1.0009	*0*.*1127*
			(0.9999–1.0017)			(0.9996–1.0021)			(0.9998–1.0019)	
	0 (1919)	111 (5.8)	1.7 (1.2–2.5)	*0*.0060*	86 (4.5)	1.2 (0.8–1.8)	*0*.*3417*	87 (4.5)	1.6 (1.1–2.4)	*0*.0282*
	1 (2117)	202 (9.5)			125 (5.9)			138 (6.5)		
Direct HDL-cholesterol (mg/dL)	4056		0.9840	*0*.0484*		0.9847	*0*.*0684*		0.9841	*0*.*0716*
			(0.9684–0.9999)			(0.9683–1.0013)			(0.9669–1.0016)	
	0 (1963)	181 (9.2)	0.6 (0.4–0.9)	*0*.0152*	115 (5.9)	0.7 (0.4–1.1)	*0*.*1257*	114 (5.8)	0.7 (0.4–1.2)	*0*.*1665*
	1 (2093)	132 (6.3)			96 (4.6)			111 (5.3)		
Total cholesterol (mg/dL)	4057		1.0003	*0*.*8095*		1.0019	*0*.*2758*		1.0050	*0*.0016*
			(0.9976–1.0030)			(0.9983–1.0056)			(1.0022–1.0078)	
	0 (1968)	146 (7.4)	1.0 (0.8–1.3)	*0*.*8837*	94 (4.8)	1.0 (0.7–1.4)	*0*.*9806*	89 (4.5)	1.4 (1.1–1.9)	*0*.0082*
	1 (2089)	167 (8.0)			117 (5.6)			136 (6.4)		
White blood cell count (1000 cells/μL)	4107		1.0796	*0*.0001*		1.0738	*0*.0172*		1.0507	*0*.0034*
			(1.0459–1.1145)			(1.0146–1.1365)			(1.0193–1.0830)	
	0 (2033)	126 (6.2)	1.7 (1.4–2.2)	*0*.0001*	94 (4.6)	1.3 (0.9–1.9)	*0*.*1054*	99 (4.9)	1.3 (1.0–1.8)	*0*.*0676*
	1 (2074)	195 (9.4)			121 (5.8)			131 (6.3)		
Platelet count SI (1000 cells/μL)	4107		1.004	*0*.*7377*		1.0012	*0*.*2094*		0.9992	*0*.*5455*
			(0.9978–1.0031)			(0.9992–1.0033)			(0.9965–1.0019)	
	0 (2183)	162 (7.4)	1.1 (0.8–1.5)	*0*.*5584*	98 (4.5)	1.4 (1.1–1.7)	*0*.0057*	111 (5.1)	1.0 (0.7–1.4)	*0*.*9109*
	1 (1924)	159 (8.3)			117 (6.1)			119 (6.2)		
C-reactive protein (mg/dL)	4068		1.1152	*0*.*0596*		1.0255	*0*.*7064*		1.0225	*0*.*7997*
			(0.9950–1.2500)			(0.8918–1.1793)			(0.8510–1.2286)	
	0 (1853)	116 (6.3)	1.4 (1.0–1.8)	*0*.0307*	*74 (3*.*4)*	1.2 (0.8–1.9)	*0*.*3100*	85 (4.6)	1.2 (0.7–2.1)	*0*.*4280*
	1 (2215)	198 (8.9)			*138 (6*.*2)*			141 (6.4)		
Cotinine (ng/mL)	4057		1.0022	*0*.0003*		1.0023	*0*.0019*		1.0013	*0*.0404*
			(1.0012–1.0032)			(1.0010–1.0036)			(1.0001–1.0025)	
	0 (2181)	139 (6.4)	1.5 (1.0–2.3)	*0*.0435*	96 (4.4)	1.9 (1.3–2.9)	*0*.0049*	107 (4.9)	1.4 (0.9–2.0)	*0*.*0942*
	1 (1876)	174 (9.3)			115 (6.1)			118 (6.3)		
Homocysteine (μmol/L)	4087		1.0319	*0*.0123*		1.0306	*0*.0491*		0.9974	*0*.*9114*
									(0.9488–1.0484)	
			(1.0079–1.0565)			(1.0001–1.0619)				
	0 (1892)	135 (7.1)	1.1 (0.9–1.4)	*0*.*3664*	79 (4.2)	1.7 (1.1–2.5)	*0*.0160*	104 (5.5)	1.1 (0.6–1.6)	*0*.*6046*
	1 (2195)	184 (8.4)			136 (6.2)			125 (5.7)		
Gamma glutamyl transferase (U/L)	4038		1.0024	*0*.*2369*		1.0022	*0*.*1330*		1.0015	*0*.*1630*
			(0.9983–1.0066)			(0.9993–1.0051)			(0.9993–1.0038)	
	0 (1868)	114 (6.1)	1.7 (1.1–2.5)	*0*.0123*	81 (4.3)	1.4 (1.0–1.9)	*0*.*0549*	91 (4.9)	1.4 (1.0–2.1)	*0*.*0663*
	1 (2170)	199 (9.2)			130 (6.0)			134 (6.2)		
Alkaline phosphatase (U/L)	4039		1.0048	*0*.*2004*		1.0044	*0*.0492*		1.0028	*0*.0473*
			(0.9972–1.0124)			(1.0000–1.0088)			(1.0000–1.0055)	
	0 (1810)	129 (7.1)	1.2 (0.7–1.9)	*0*.*5499*	80 (4.4)	1.2 (0.9–1.6)	*0*.*1602*	91 (5.0)	1.1 (0.8–1.6)	*0*.*4106*
	1 (2229)	184 (8.2)			131 (5.9)			134 (6.0)		
Total calcium (mg/dL)	4039		0.9301	*0*.*7475*		1.2505	*0*.*3030*		1.2478	*0*.*2703*
			(0.5808–1.4896)			(0.8000–1.9547)			(0.8262–1.8846)	
	0 (1701)	126 (7.4)	1.0 (0.7–1.4)	*0*.*8952*	77 (4.5)	1.4 (1.0–2.0)	*0*.*0771*	82 (4.8)	1.4 (1.0–2.1)	*0*.*0642*
	1 (2338)	187 (8.0)			134 (5.7)			143 (6.1)		
Bicarbonate (mmol/L)	4039		1.0766	*0*.*0652*		1.063	*0*.*1286*		1.0656	*0*.0413*
			(0.9947–1.1653)			(0.9788–1.1659)			(1.0029–1.1323)	
	0 (1712)	117 (6.8)	1.4 (1.0–2.0)	*0*.*0819*	80 (4.7)	1.3 (1.0–1.8)	*0*.*0827*	86 (5.0)	1.3 (1.0–1.8)	*0*.*0611*
	1 (2327)	196 (8.4)			131 (5.6)			139 (6.0)		
Lactate dehydrogenase LDH (U/L)	4035		1.002	*0*.*9331*		1.0025	*0*.0374*		0.9999	*0*.*9715*
			(0.9959–1.0045)			(1.0002–1.0048)			(0.9959–1.0039)	
	0 (1872)	138 (7.4)	1.0 (0.7–1.4)	*0*.*9720*	86 (4.6)	1.0 (0.8–1.3)	*0*.*7500*	98 (5.2)	0.9 (0.7–1.3)	*0*.*6447*
	1 (2163)	175 (8.1)			125 (5.8)			127 (5.9)		
Phosphorus (mg/dL)	4038		1.0239	*0*.*8620*		1.1541	*0*.*3135*		1.1518	*0*.*3542*
			(0.7703–1.3609)			(0.8611–1.5469)			(0.8405–1.5782)	
	0 (1929)	159 (8.2)	1.0 (0.7–1.2)	*0*.*6931*	106 (5.5)	1.0 (0.8–1.4)	*0*.*7803*	107 (5.6)	1.2 (0.9–1.5)	*0*.*2498*
	1 (2109)	154 (7.3)			105 (5.0)			118 (5.6)		
Total protein (g/L)	4034		0.6798	*0*.0098*		0.9473	*0*.*7561*		0.6611	*0*.0363*
			(0.5147–0.8980)			(0.6575–1.3647)			(0.4504–0.9702)	
	0 (1882)	174 (9.3)	0.6 (0.5–0.9)	*0*.0186*	107 (5.7)	0.8 (0.6–1.2)	*0*.*2698*	125 (6.6)	0.7 (0.5–1.1)	*0*.*0857*
	1 (2155)	139 (6.5)			104 (4.8)			100 (4.6)		
Uric acid (mg/dL)	4037		1.0212	*0*.*7779*		1.0181	*0*.*8321*		0.9058	*0*.*2573*
			(0.8741–1.1931)			(0.8526–1.2158)			(0.7572–1.0835)	
	0 (1970)	156 (7.9)	0.9 (0.7–1.2)	*0*.*4857*	114 (5.8)	0.8 (0.5–1.1)	*0*.*1256*	129 (6.6)	0.8 (0.5–1.2)	*0*.*2412*
	1 (2067)	157 (7.6)			97 (4.7)			96 (4.6)		
Sodium (mmol/L)	4039		0.9521	*0*.*1789*		0.9720	*0*.*4736*		1.0065	*0*.*8384*
						(0.8952–1.0554)			(0.9415–1.0760)	
			(0.8839–1.0254)							
	0 (1505)	177 (10.6)	0.9 (0.6–1.3)	*0*.*5992*	66 (4.4)	1.3 (0.8–2.0)	*0*.*2300*	76 (5.1)	1.3 (0.9–1.9)	*0*.*1614*
	1 (2534)	189 (7.5)			145 (5.7)			149 (5.9)		
Potassium (mmol/L)	4038		1.4825	*0*.*2280*		1.4903	*0*.*1801*		2.2785 (1.3978–3.7141)	*0*.0027*
			(0.7604–2.8906)			(0.8139–2.7290)				
	0 (1850)	151 (8.2)	1.0 (0.6–1.5)	*0*.*9827*	98 (5.3)	1.0 (0.8–1.4)	*0*.*7982*	103 (5.6)	1.2 (0.8–1.8)	*0*.*3309*
	1 (2188)	162 (7.4)			113 (5.2)			122 (5.6)		
Chloride (mmol/L)	4039		0.9184	*0*.0320*		0.9476	*0*.*2337*		0.9855	*0*.*6737*
			(0.8505–0.9917)			(0.8640–1.0394)			(0.9165–1.0597)	
	0 (1826)	164 (9.0)	0.7 (0.5–1.1)	*0*.*1095*	109 (6.0)	0.7 (0.5–1.1)	*0*.*1263*	108 (5.9)	1.0 (0.7–1.4)	*0*.*8701*
	1 (2213)	149 (6.7)			102 (4.6)			117 (5.3)		
Globulin (g/dL)	4037		0.7779	*0*.*1082*		0.8244	*0*.*2628*		0.6230	*0*.0336*
			(0.5687–1.0642)			(0.5787–1.1743)			(0.40048–0.9589)	
	0 (1593)	134 (8.4)	0.8 (0.6–1.2)	*0*.*2899*	91 (5.7)	0.9 (0.6–1.2)	*0*.*3529*	107 (6.7)	0.7 (0.5–0.9)	*0*.0153*
	1 (2444)	179 (7.3)			120 (4.9)			118 (4.8)		

^a^ Chronic pain as pain that persists beyond normal tissue healing time, which is assumed to be 3 months. Participants with pain lasting ≥24 hours in the past month at one of the anatomical sites studied were asked for how long they experienced this pain: ≤1 month, between 1 and 3 months, at least 3 months but less than 1 year or ≥1 year. Participants who answered “at least 3 months but less than 1 year and ≥1 year” were considered to have chronic pain.

^b^ All analyses adjusted for sex (male, female), age (20–34; 35–49; 50–64; 65–79; ≥80 years), and BMI (<20; 20–24.9; 25–29.9; ≥30).

Associations are presented for biomarkers considered continuously and dichotomized at the median of the distribution.

^c^ Since NHANES data were weighted to make them comparable to those of the non-institutionalized US population, the proportions are not exactly 50% on each side of the median.

In separate analyses on acute and subacute pain, very few biomarkers were statistically associated with the outcomes; more importantly, the _a_OR was rarely far from 1.0. Only white blood cell count (_a_OR: 1.9, 95%CI: 1.0–3.7), alkaline phosphatase (_a_OR: 0.5, 95%CI: 0.3–0.9) and gamma glutamyl transferase (_a_OR: 2.5, 95%CI: 1.2–5.3) were statistically associated with acute neck pain (Table 4 in S1 Appendix). For subacute pain, β-carotene (_a_OR: 0.2, 95%CI: 0.1–0.4) and total mercury (_a_OR: 0.3, 95%CI: 0.1–0.8) were associated with shoulder pain, total cholesterol (_a_OR: 3.1, 95%CI: 1.1–8.4) and gamma glutamyl transferase (_a_OR: 4.1, 95%CI: 1.2–13.8) were associated with neck pain, and bicarbonate (_a_OR: 0.3, 95%CI: 0.1–0.8) was associated with low back pain (Table 5 in S2 Appendix). For both acute and subacute pain, no biomarkers were statistically associated with two or three sites.

Results were similar when biomarkers were categorized based on quartiles: almost all the variables associated with at least two pain sites in the main analyses retained their statistical significance.

## Discussion

This study used unique data to explore the associations of numerous biomarkers with self-reported musculoskeletal pain on three anatomical sites. The determinants of musculoskeletal pain are undoubtedly biopsychosocial, but very few studies have examined other potential biological determinants than clinical and mechanical ones. Our study thus makes an original contribution by providing the first report examining the relationship of several dozens of biomarkers with musculoskeletal pain. Only a few positive and inverse associations between biomarkers and chronic musculoskeletal pain syndromes were identified.

The literature on biomarkers of musculoskeletal pain is quite scarce. A recent systematic review found moderate evidence for a direct association between pro-inflammatory biomarkers, tumor necrosis factor alpha (TNF-α), C-reactive protein, interleukin-6, and nonspecific low back pain [21]. Although this evidence is important, the investigators only looked at one site of musculoskeletal pain with a limited number of biomarkers. While their conclusion on C-reactive protein being statistically associated with low back pain is consistent with our findings, we used very stringent criteria to retain biomarkers, including a statistically significant association with at least two pain sites, and C-reactive protein did not qualify (although it was significant for low back pain). This approach was chosen to allow the identification of only the most prominent markers among many, but in so doing, could have resulted in missing ones.

Croft et al. [22], Natvig et al. [23], and Thomas et al. [24] found that musculoskeletal pain at one site is often accompanied by pain at other sites. Our finding of a few biomarkers being associated with chronic pain in at least two sites is consistent with the hypothesis of a systemic mechanism for chronic musculoskeletal pain.

Some authors have noted significant associations between specific vitamins and fibromyalgia [25]. In other studies, although unclear for α-carotene [26], while plasma levels of provitamin A, β-carotene, were inversely associated with higher risk of fracture [27], other associations were direct; for instance, increased risk of fracture at high serum concentrations of retinol (s-retinol) has been observed in epidemiologic studies [28, 29]. More recently, cumulative dose of isotretinoin, a vitamin A derivative used to treat acne, has been associated with low back pain [30]. Ascorbic acid (vitamin C) for its part, is essential for the activity of some enzymes (proline hydroxylase and lysine hydroxylase) that are necessary to maintain stable collagen helixes that characterize healthy connective tissues [31]. In a previous report, suboptimal serum ascorbic acid concentrations were found to be independently associated with the prevalence of neck pain, low back pain, and low back pain with pain below knee in the past three months, self-reported diagnosis of arthritis/rheumatism and related functional limitations [32].

Exposure to environmental cadmium poses many public health problems, as it is a highly toxic substance that can cause important adverse health effects [33–35]. At high doses, cadmium is known to cause the so-called *itai-itai* disease whose dominant symptom is back pain [36]. Like isotretinoin [37], cadmium is also known to be positively associated with homocysteine [38, 39], alkaline phosphatase [40], white blood cell count [41], triglycerides and monocyte elevation [42], HDL-cholesterol [43–45] and total protein reduction [44, 46]. Triglycerides are positively associated with retinol and α-tocopherol but negatively associated with β-carotene [45], which is partly consistent with our findings. Chronic mercury exposure has devastating effects on the human body [47], although mercury has been used for long time for its medicinal properties, including as a diuretic, antibacterial agent and laxative [48]. Our results showing a protective association between mercury and chronic pain may be spurious, as observed in some studies [49], or it may represent a proxy for fish consumption as in others [50]. Actually, 99% of participants showed mercury levels much below 20 μg/L, which is considered normal.

Cotinine is a marker of smoking that is preferred to nicotine because of its longer half-life [51]. The association of smoking with musculoskeletal pain is very consistent and follows a dose-response gradient. This relationship is not explained by vitamin C deficiency among smokers [52]. Acrylamide, a marker that has been classified as a probable carcinogen, can be ingested, inhaled (in tobacco smoke) or absorbed [28]. It is formed in high amounts in many types of food prepared at high temperature. It has also been shown to cause progressive peripheral neuropathy [53]. Our results that cotinine, acrylamide and its metabolite glycidamide [54], and cadmium—all cigarette smoke derivatives—being positively associated with chronic musculoskeletal pain, are compatible with the hypothesis placing them as mediators of the positive association between smoking and musculoskeletal pain; this hypothesis will need to be tested in future work.

While our study was exploratory, the fact that most associations identified were with chronic musculoskeletal pain and not with acute or subacute syndromes is interesting and again, would support the hypothesis of systemic mechanisms for chronic musculoskeletal pain.

The NHANES survey included a large national sample that was representative of the non-institutionalized population, which suggests that a selection bias is unlikely to have affected our results. Also, NHANES offered specific laboratory information on almost 200 biomarkers, using rigorous quality control and measurement instruments that provided high-quality data, a rare opportunity. The large number of participants provided high statistical power and precise estimates. We used a conservative and systemic approach to diminish chance findings.

Among the study limitations, its cross-sectional design does not allow to conclude to causal associations. Also, because musculoskeletal pain was self-reported, it is possible that our measures of outcomes suffered from non-differential misclassification due to recall bias. However, since the reference period was short, such a recall bias is probably limited. The definitions of musculoskeletal pain often differ between studies; we used definitions similar to those published by Dionne et al. [55] and Griffith et al. [56] that are widely used. These definitions require that participants had experienced pain during the past four weeks. Such definitions minimize recall bias since the focus is not on pain duration and the measurement instruments have been validated. Non-differential misclassification of the biomarkers is also to be expected, since each biomarker has a different threshold of exposure. Given the number of markers examined and the exploratory nature of our study, we could not use specific threshold for each marker. While the fact that most associations were found with chronic pain supports biological plausibility, the smaller sample sizes in the acute and subacute groups and the consequent limited statistical power could have prevented us to identify important associations in these subgroups. Finally, residual confounding is likely, given the same basic adjustments we have made in all regression models. Future studies on individual markers will be able to identify specific confounders and adjust for them, an enterprise that was impossible here given the numerous markers examined. Again, in interpreting the results of this study, it is thus important to keep its exploratory nature in mind.

## Conclusions

In these exploratory stringent analyses of a very unique set of data, we found strong and consistent associations between some biomarkers and chronic musculoskeletal pain in the low back, shoulder, and neck. Acrylamide, glycidamide, α-carotene, β-carotene, cadmium, cotinine, mercury, retinol (vitamin A), triglycerides, white blood cell, homocysteine, alkaline phosphatase, total protein and ascorbic acid (vitamin C) had the strongest and more consistent associations with chronic musculoskeletal pain. As knowledge on the determinants of musculoskeletal pain is still very limited, these results could have tremendous implications in the field by opening new avenues of research. Research on musculoskeletal pain needs to put more effort on the biological dimension of the biopsychosocial model of pain.

## Supporting information

S1 Appendix Table 4. Results of multivariable analyses of the associations between biomarkers retained and the three acute pain sites studied (n = 3,834). (DOCX)

S2 Appendix Table 5. Results of multivariable analyses of the associations between biomarkers retained and the three subacute pain sites studied (n = 3,658). (DOCX)

S3 Appendix List of specific biomarkers considered in NHANES 2003–2004. (DOCX)

S1 Data (ZIP)

## References

[1] Hoy DG, Smith E, Cross M, Sanchez-Riera L, Buchbinder R, Blyth FM, et al. The global burden of musculoskeletal conditions for 2010: an overview of methods. Ann Rheum Dis. 2014;73(6):982–9. Epub 2014/02/20. doi: 10.1136/annrheumdis-2013-204344 . 24550172

[2] Maher C, Underwood M, Buchbinder R. Non-specific low back pain. Lancet. 2017;389(10070):736–47. doi: 10.1016/S0140-6736(16)30970-9 . 27745712

[3] Murray CJ, Vos T, Lozano R, Naghavi M, Flaxman AD, Michaud C, et al. Disability-adjusted life years (DALYs) for 291 diseases and injuries in 21 regions, 1990–2010: a systematic analysis for the Global Burden of Disease Study 2010. Lancet. 2012;380(9859):2197–223. doi: 10.1016/S0140-6736(12)61689-4 . 23245608

[4] Hoy D, March L, Brooks P, Blyth F, Woolf A, Bain C, et al. The global burden of low back pain: estimates from the Global Burden of Disease 2010 study. Ann Rheum Dis. 2014;73(6):968–74. doi: 10.1136/annrheumdis-2013-204428 . 24665116

[5] Waddell G. 1987 Volvo award in clinical sciences. A new clinical model for the treatment of low-back pain. Spine (Phila Pa 1976). 1987;12(7):632–44. Epub 1987/09/01. doi: 10.1097/00007632-198709000-00002 . 2961080

[6] Buchbinder R, van Tulder M, Oberg B, Costa LM, Woolf A, Schoene M, et al. Low back pain: a call for action. Lancet. 2018;391(10137):2384–8. doi: 10.1016/S0140-6736(18)30488-4 . 29573871

[7] Puntmann VO. How-to guide on biomarkers: biomarker definitions, validation and applications with examples from cardiovascular disease. Postgrad Med J. 2009;85(1008):538–45. Epub 2009/10/01. doi: 10.1136/pgmj.2008.073759 . 19789193

[8] Johnson CL, Paulose-Ram R, Ogden CL, Carroll MD, Kruszon-Moran D, Dohrmann SM, et al. National health and nutrition examination survey: analytic guidelines, 1999–2010. Vital Health Stat 2. 2013;(161):1–24. Epub 2014/08/05. . 25090154

[9] Ahluwalia N, Dwyer J, Terry A, Moshfegh A, Johnson C. Update on NHANES Dietary Data: Focus on Collection, Release, Analytical Considerations, and Uses to Inform Public Policy. Adv Nutr. 2016;7(1):121–34. Epub 2016/01/17. doi: 10.3945/an.115.009258 . 26773020 PMC4717880

[10] Curtin LR, Mohadjer LK, Dohrmann SM, Montaquila JM, Kruszan-Moran D, Mirel LB, et al. The National Health and Nutrition Examination Survey: Sample Design, 1999–2006. Vital Health Stat 2. 2012;(155):1–39. Epub 2012/07/14. . 22788053

[11] Treede RD, Rief W, Barke A, Aziz Q, Bennett MI, Benoliel R, et al. Chronic pain as a symptom or a disease: the IASP Classification of Chronic Pain for the International Classification of Diseases (ICD-11). Pain. 2019;160(1):19–27. doi: 10.1097/j.pain.0000000000001384 . 30586067

[12] Elliott AM, Smith BH, Penny KI, Smith WC, Chambers WA. The epidemiology of chronic pain in the community. Lancet. 1999;354(9186):1248–52. doi: 10.1016/s0140-6736(99)03057-3 . 10520633

[13] Blyth FM, Briggs AM, Schneider CH, Hoy DG, March LM. The Global Burden of Musculoskeletal Pain-Where to From Here? Am J Public Health. 2019;109(1):35–40. Epub 2018/11/30. doi: 10.2105/AJPH.2018.304747 . 30495997 PMC6301413

[14] NHANES, NHANES 2003–2004 Laboratory Methods, https://wwwn.cdc.gov/nchs/nhanes/ContinuousNhanes/LabMethods.aspx?BeginYear=2003. In view of Oct. 2020.

[15] Douketis JD, Paradis G, Keller H, Martineau C. Canadian guidelines for body weight classification in adults: application in clinical practice to screen for overweight and obesity and to assess disease risk. CMAJ. 2005;172(8):995–8. Epub 2005/04/13. doi: 10.1503/cmaj.045170 . 15824401 PMC556034

[16] Flegal KM, Kit BK, Orpana H, Graubard BI. Association of all-cause mortality with overweight and obesity using standard body mass index categories: a systematic review and meta-analysis. JAMA. 2013;309(1):71–82. Epub 2013/01/03. doi: 10.1001/jama.2012.113905 . 23280227 PMC4855514

[17] Centers for Disease Control and Prevention (CDC). National Center for Health Statistics (NCHS). Examination Survey Analytic and Reporting Guidelines. Hyattsville, MD: U.S. Department of Health and Human Services, Centers for Disease Control and Prevention, Sept. 2006. http://www.cdc.gov/nchs/data/nhanes/nhanes_03_04/nhanes_analytic_guidelines_dec_2005.pdf (cited Oct. 20, 2010). CDC/National Center for Health Statistics; 2006.

[18] SAS Institute Inc. The SAS System for Sun OS. Cary NSII.

[19] Rothman KJ. No adjustments are needed for multiple comparisons. Epidemiology. 1990;1(1):43–6. Epub 1990/01/01. . 2081237

[20] Rubin M. When to Adjust Alpha During Multiple Testing: A Consideration of Disjunction, Conjunction, and Individual Testing. Synthese 2021. doi: 10.1007/s11229-021-03276-4

[21] van den Berg R, Jongbloed EM, de Schepper EIT, Bierma-Zeinstra SMA, Koes BW, Luijsterburg PAJ. The association between pro-inflammatory biomarkers and nonspecific low back pain: a systematic review. Spine J. 2018;18(11):2140–51. Epub 2018/07/01. doi: 10.1016/j.spinee.2018.06.349 . 29960111

[22] Croft P, Jordan K, Jinks C. "Pain elsewhere" and the impact of knee pain in older people. Arthritis Rheum. 2005;52(8):2350–4. doi: 10.1002/art.21218 . 16052574

[23] Natvig B, Bruusgaard D, Eriksen W. Localized low back pain and low back pain as part of widespread musculoskeletal pain: two different disorders? A cross-sectional population study. J Rehabil Med. 2001;33(1):21–5. doi: 10.1080/165019701300006498 11480465

[24] Thomas E, Silman AJ, Croft PR, Papageorgiou AC, Jayson MI, Macfarlane GJ. Predicting who develops chronic low back pain in primary care: a prospective study. Bmj. 1999;318(7199):1662–7. doi: 10.1136/bmj.318.7199.1662 10373170 PMC28145

[25] Batista ED, Andretta A, de Miranda RC, Nehring J, Dos Santos Paiva E, Schieferdecker ME. Food intake assessment and quality of life in women with fibromyalgia. Rev Bras Reumatol Engl Ed. 2016;56(2):105–10. Epub 2016/06/09. doi: 10.1016/j.rbre.2015.08.015 . 27267522

[26] Cao WT, Zeng FF, Li BL, Lin JS, Liang YY, Chen YM. Higher dietary carotenoid intake associated with lower risk of hip fracture in middle-aged and elderly Chinese: A matched case-control study. Bone. 2018;111:116–22. doi: 10.1016/j.bone.2018.03.023 . 29605302

[27] Hayhoe RPG, Lentjes MAH, Mulligan AA, Luben RN, Khaw KT, Welch AA. Carotenoid dietary intakes and plasma concentrations are associated with heel bone ultrasound attenuation and osteoporotic fracture risk in the European Prospective Investigation into Cancer and Nutrition (EPIC)-Norfolk cohort. Br J Nutr. 2017;117(10):1439–53. Epub 2017/06/08. doi: 10.1017/S0007114517001180 . 28587685

[28] Baineni R, Gulati R, Delhi CK. Vitamin A toxicity presenting as bone pain. Arch Dis Child. 2017;102(6):556–8. Epub 2016/06/09. doi: 10.1136/archdischild-2016-310631 . 27272974

[29] Penniston KL, Tanumihardjo SA. The acute and chronic toxic effects of vitamin A. Am J Clin Nutr. 2006;83(2):191–201. Epub 2006/02/14. doi: 10.1093/ajcn/83.2.191 . 16469975

[30] Karaosmanoglu N, Mulkoglu C. Analysis of musculoskeletal side effects of oral Isotretinoin treatment: a cross-sectional study. BMC Musculoskelet Disord. 2020;21(1):631. Epub 2020/09/27. doi: 10.1186/s12891-020-03656-w . 32977793 PMC7519514

[31] Abdullah M, Jamil RT, Attia FN. Vitamin C (Ascorbic Acid). StatPearls. Treasure Island (FL)2020.

[32] Dionne CE, Laurin D, Desrosiers T, Abdous B, Le Sage N, Frenette J, et al. Serum vitamin C and spinal pain: a nationwide study. Pain. 2016;157(11):2527–35. Epub 2016/10/19. doi: 10.1097/j.pain.0000000000000671 . 27434504

[33] Faroon O, Ashizawa A, Wright S, Tucker P, Jenkins K, Ingerman L, et al. Toxicological Profile for Cadmium. Agency for Toxic Substances and Disease Registry (ATSDR) Toxicological Profiles. Atlanta (GA)2012. 24049863

[34] La-Up A, Wiwatanadate P, Uthaikhup S, Pruenglampoo S. Association between urinary cadmium and chronic musculoskeletal pain in residents of cadmium-contaminated area in Northwest Thailand. Environ Sci Pollut Res Int. 2018;25(14):14182–7. Epub 2018/03/11. doi: 10.1007/s11356-018-1665-3 . 29524173

[35] Satarug S, Garrett SH, Sens MA, Sens DA. Cadmium, environmental exposure, and health outcomes. Cien Saude Colet. 2011;16(5):2587–602. Epub 2011/06/10. doi: 10.1590/s1413-81232011000500029 . 21655733 PMC5967636

[36] Inaba T, Kobayashi E, Suwazono Y, Uetani M, Oishi M, Nakagawa H, et al. Estimation of cumulative cadmium intake causing Itai-itai disease. Toxicol Lett. 2005;159(2):192–201. Epub 2005/07/12. doi: 10.1016/j.toxlet.2005.05.011 . 16006079

[37] Lee YH, Scharnitz TP, Muscat J, Chen A, Gupta-Elera G, Kirby JS. Laboratory Monitoring During Isotretinoin Therapy for Acne: A Systematic Review and Meta-analysis. JAMA Dermatol. 2016;152(1):35–44. Epub 2015/12/03. doi: 10.1001/jamadermatol.2015.3091 . 26630323

[38] Polat M, Lenk N, Bingol S, Oztas P, Ilhan MN, Artuz F, et al. Plasma homocysteine level is elevated in patients on isotretinoin therapy for cystic acne: a prospective controlled study. J Dermatolog Treat. 2008;19(4):229–32. doi: 10.1080/09546630701846079 . 18608712

[39] Guallar E, Silbergeld EK, Navas-Acien A, Malhotra S, Astor BC, Sharrett AR, et al. Confounding of the relation between homocysteine and peripheral arterial disease by lead, cadmium, and renal function. Am J Epidemiol. 2006;163(8):700–8. doi: 10.1093/aje/kwj090 . 16484446

[40] Kang MY, Cho SH, Lim YH, Seo JC, Hong YC. Effects of environmental cadmium exposure on liver function in adults. Occup Environ Med. 2013;70(4):268–73. doi: 10.1136/oemed-2012-101063 . 23322921

[41] Ma S, Zhang J, Xu C, Da M, Xu Y, Chen Y, et al. Increased serum levels of cadmium are associated with an elevated risk of cardiovascular disease in adults. Environ Sci Pollut Res Int. 2021. doi: 10.1007/s11356-021-15732-2 . 34363163

[42] Karakaya A, Yucesoy B, Sardas OS. An immunological study on workers occupationally exposed to cadmium. Hum Exp Toxicol. 1994;13(2):73–5. Epub 1994/02/01. doi: 10.1177/096032719401300202 . 7908813

[43] Lovasova E, Racz O, Cimbolakova I, Novakova J, Dombrovsky P, Nistiar F. Effects of chronic low-dose cadmium exposure on selected biochemical and antioxidant parameters in rats. J Toxicol Environ Health A. 2013;76(17):1033–8. doi: 10.1080/15287394.2013.828249 . 24168039

[44] Samarghandian S, Azimi-Nezhad M, Shabestari MM, Azad FJ, Farkhondeh T, Bafandeh F. Effect of chronic exposure to cadmium on serum lipid, lipoprotein and oxidative stress indices in male rats. Interdiscip Toxicol. 2015;8(3):151–4. Epub 2016/08/04. doi: 10.1515/intox-2015-0023 . 27486375 PMC4961912

[45] Ireland P, Jolley D, Giles G, Powles J, O’Dea K, Hopper J, et al. Determinants of serum levels of retinol, beta-carotene and alpha-tocopherol in men and women born in Australia, Greece and Italy. Asia Pac J Clin Nutr. 1994;3(4):169–77. . 24351327

[46] Rogalska J, Brzoska MM, Roszczenko A, Moniuszko-Jakoniuk J. Enhanced zinc consumption prevents cadmium-induced alterations in lipid metabolism in male rats. Chem Biol Interact. 2009;177(2):142–52. Epub 2008/10/14. doi: 10.1016/j.cbi.2008.09.011 . 18848534

[47] Carter JA, Desai SM, Probst J, Kogan M. Integrative Medicine Approach To Peripheral Neuropathy-Avoiding Pitfalls Of Ineffective Current Standards In Assessing Chronic Low-Grade Mercury Toxicity And Functional Musculoskeletal Lesions. Integr Med (Encinitas). 2019;18(5):49–55. Epub 2020/06/19. . 32549846 PMC7219441

[48] Masur LC. A review of the use of mercury in historic and current ritualistic and spiritual practices. Altern Med Rev. 2011;16(4):314–20. Epub 2012/01/05. . 22214251

[49] Kroger E, Verreault R, Carmichael PH, Lindsay J, Julien P, Dewailly E, et al. Omega-3 fatty acids and risk of dementia: the Canadian Study of Health and Aging. Am J Clin Nutr. 2009;90(1):184–92. Epub 2009/05/29. doi: 10.3945/ajcn.2008.26987 . 19474137

[50] Petrova MV, Ourgaud M, Boavida JRH, Dufour A, Tesan Onrubia JA, Lozingot A, et al. Human mercury exposure levels and fish consumption at the French Riviera. Chemosphere. 2020;258:127232. Epub 2020/06/17. doi: 10.1016/j.chemosphere.2020.127232 . 32540539

[51] Jarvis MJ, Russell MA, Benowitz NL, Feyerabend C. Elimination of cotinine from body fluids: implications for noninvasive measurement of tobacco smoke exposure. Am J Public Health. 1988;78(6):696–8. doi: 10.2105/ajph.78.6.696 3369603 PMC1350287

[52] Dionne CE, Laurin D, Desrosiers T, Abdous B, Le Sage N, Frenette J, et al. Vitamin C is not the Missing Link Between Cigarette Smoking and Spinal Pain. Spine (Phila Pa 1976). 2018;43(12):E712–E21. Epub 2017/12/15. doi: 10.1097/BRS.0000000000002466 . 29239908

[53] Spencer PS, Schaumburg HH. Nervous system degeneration produced by acrylamide monomer. Environ Health Perspect. 1975;11:129–33. Epub 1975/06/01. doi: 10.1289/ehp.7511129 170076 PMC1475186

[54] Pruser KN F N. Acrylamide in health and disease. Front Biosci (Schol Ed). 2011;3:41–51. Epub Epub 2011/01/05. doi: 10.2741/s130 . 21196355

[55] Dionne CE, Dunn KM, Croft PR, Nachemson AL, Buchbinder R, Walker BF, et al. A consensus approach toward the standardization of back pain definitions for use in prevalence studies. Spine (Phila Pa 1976). 2008;33(1):95–103. Epub 2008/01/01. doi: 10.1097/BRS.0b013e31815e7f94 . 18165754

[56] Griffith LE, Hogg-Johnson S, Cole DC, Krause N, Hayden J, Burdorf A, et al. Low-back pain definitions in occupational studies were categorized for a meta-analysis using Delphi consensus methods. J Clin Epidemiol. 2007;60(6):625–33. Epub 2007/05/12. doi: 10.1016/j.jclinepi.2006.09.005 . 17493522

