# Supplementary material for: An exploratory identification of biological markers of chronic musculoskeletal pain in the low back, neck, and shoulders
Source: PLoS One. 2022 Apr 15;17(4):e0266999. doi: 10.1371/journal.pone.0266999 (PMC9012384; doi:10.1371/journal.pone.0266999)
Supplement: S1 Appendix — (DOCX) [file pone.0266999.s001.docx]

**APPENDIX 1:**

**Table 4. Results of multivariable analyses of the associations between biomarkers retained and the three acute pain^a^ sites studied (n = 3,834^b^).**

| **Biomarkers^c^** | **Class (n)^d^** | **Low back pain** | | | **Shoulder pain** | | | **Neck pain** | | |
| --- | --- | --- | --- | --- | --- | --- | --- | --- | --- | --- |
|  |  | **Frequency by class (%)** | **Odds Ratio (IC - 95%)** | ***P value*** | **Frequency by class (%)** | **Odds Ratio (IC - 95%)** | ***P value*** | **Frequency by class (%)** | **Odds Ratio (IC - 95%)** | ***P value*** |
| Acrylamide (pmoL/G Hb) | 0 (1752) | 31 (1.2) | 1.6 (0.9 – 3.0) | *0.1150* | 16 (0.9) | 1.0 (0.4 – 2.3) | *0.8239* | 20 (1.1) | 0.7 (0.4 – 1.3) | *0.2647* |
|  | 1 (1544) | 43 (1.3) |  |  | 18 (1.2) |  |  | 20 (1.3) |  |  |
| Glycidamide (pmoL/G Hb) | 0 (1773) | 30 (0.9) | 1.6 (0.8 – 2.9) | *0.1457* | 14 (0.0) | 1.0 (0.4 – 2.7) | *0.9883* | 20 (1.1) | 1.0 (0.5 – 2.0) | *0.9772* |
|  | 1 (1574) | 43 (1.3) |  |  | 19 (1.2) |  |  | 24 (1.5) |  |  |
| Albumin, urine (ug/mL) | 0 (1665) | 32 (1.9) | 1.2 (0.6 – 2.4) | *0.5615* | 17 (1.02) | 1.1 (0.5 – 2.4) | *0.8448* | 22 (1.3) | 0.8 (0.4 – 1.8) | *0.5986* |
|  | 1 (2055) | 49 (2.4) |  |  | 21 (1.0) |  |  | 26 (1.3) |  |  |
| Ascorbic acid  (µmol/L)  (Vitamin C) | 0 (1723) | 41 (2.4) | 1.0 (0.4 – 2.5) | *0.9836* | 20 (1.2) | 1.1 (0.4 – 2.8) | *0.8650* | 32 (1.9) | 0.7 (0.3 – 1.7) | *0.3646* |
|  | 1 (1863) | 35 (1.9) |  |  | 16 (0.9) |  |  | 17 (0.9) |  |  |
| Retinol (µg/dL) (Vitamin A) | 0 (1985) | 46 (2.3) | 1.0 (0.5 – 1.8) | *0.9206* | 17 (0.9) | 1.5 (0.7 – 3.1) | *0.2609* | 29 (1.5) | 1.0 (0.5 – 1.9) | *0.9908* |
|  | 1 (1614) | 31 (1.9) |  |  | 20 (1.2) |  |  | 20 (1.2) |  |  |
| α-Tocopherol (µg/dL)  (Vitamin E) | 0 (1788) | 46 (2.6) | 0.9 (0.5 – 1.7) | *0.8329* | 25 (1.4) | 0.7 (0.3 – 1.4) | *0.2812* | 30 (1.7) | 1.1 (0.6 – 2.1) | *0.6939* |
|  | 1 (1811) | 31 (1.7) |  |  | 12 (0.7) |  |  | 19 (1.1) |  |  |
| α-Carotene (µg/dL) | 0 (1667) | 41 (2.5) | 0.8 (0.4 – 1.6) | *0.5127* | 18 (1.1) | 1.2 (0.5 – 2.7) | *0.6967* | 29 (1.7) | 0.9 (0.5 – 1.8) | *0.8077* |
|  | 1 (1931) | 36 (1.9) |  |  | 19 (1.0) |  |  | 20 (1.0) |  |  |
| β-Carotene (µg/dL) | 0 (1660) | 44 (2.7) | 0.8 (0.5 – 1.4) | *0.3640* | 21 (1.3) | 0.9 (0.5 – 1.6) | *0.7907* | 28 (1.7) | 1.0 (0.6 – 1.6) | *0.9236* |
|  | 1 (1939) | 33 (1.7) |  |  | 16 (0.8) |  |  | 21 (1.1) |  |  |
| Cadmium (µg/L) | 0 (1725) | 45 (2.6) | 1.0 (0.6 – 1.6) | *0.8716* | 17 (1.0) | 1.1 (0.4 – 3.0) | *0.8267* | 24 (1.4) | 1.2 (0.5 – 2.6) | *0.6514* |
|  | 1 (1925) | 33 (1.7) |  |  | 21 (1.1) |  |  | 26 (1.4) |  |  |
| Lead (µg/dL) | 0 (1578) | 43 (2.7) | 1.0 (0.5 – 1.9) | *0.9866* | 16 (1.0) | 1.1 (0.4 – 3.0) | *0.8232* | 29 (1.8) | 0.7 (0.3 – 1.9) | *0.5144* |
|  | 1 (2072) | 35 (1.7) |  |  | 22 (1.1) |  |  | 21 (1.0) |  |  |
| Mercury, total (µg/L) | 0 (1830) | 41 (2.2) | 0.8 (0.4 – 1.6) | *0.5875* | 14 (0.8) | 1.5 (0.6 – 3.9) | *0.3562* | 26 (1.4) | 0.8 (0.3 – 2.0) | *0.6030* |
|  | 1 (1820) | 37 (2.0) |  |  | 24 (1.3) |  |  | 24 (1.3) |  |  |
| Triglycerides (mg/dL) | 0 (1765) | 40 (2.3) | 1.0 (0.6 – 1.6) | *0.9599* | 16 (0.9) | 0.9 (0.4 – 2.3) | *0.8572* | 22 (1.3) | 1.1 (0.6 – 1.9) | *0.7283* |
|  | 1 (1832) | 37 (2.0) |  |  | 21 (1.2) |  |  | 27 (1.5) |  |  |
| Direct HDL-cholesterol (mg/dL) | 0 (1715) | 42 (2.5) | 1.0 (0.6 – 1.6) | *0.9150* | 22 (1.3) | 0.6 (0.3 – 1.4) | *0.2633* | 29 (1.7) | 0.7 (0.4 – 1.2) | *0.3987* |
|  | 1 (1900) | 35 (1.8) |  |  | 15 (0.8) |  |  | 20 (1.1) |  |  |
| Total cholesterol (mg/dL) | 0 (1764) | 42 (2.4) | 1.0 (0.7 – 1.6) | *0.8225* | 18 (1.0) | 1.0 (0.5 – 2.0) | *0.8857* | 22 (1.3) | 1.5 (0.8 – 3.0) | *0.1811* |
|  | 1 (1852) | 35 (1.9) |  |  | 19 (1.0) |  |  | 27 (1.5) |  |  |
| White blood cell count (1000 cells/µL) | 0 (1845) | 40 (2.2) | 1.0 (0.5 – 1.8) | *0.9402* | 15 (0.8) | 1.9 (0.7 – 4.9) | *0.1989* | 16 (0.9) | 1.9 (1.0 – 3.7) | *0.0487** |
|  | 1 (1809) | 38 (2.1) |  |  | 23 (1.3) |  |  | 34 (1.9) |  |  |
| Platelet count SI (1000 cells/µL) | 0 (1929) | 36 (1.9) | 0.9 (0.4 – 2.1) | *0.8595* | 19 (1.0) | 0.8 (0.3 – 2.1) | *0.6886* | 18 (0.9) | 1.5 (0.9 – 2.7) | *0.0924* |
|  | 1 (1725) | 42 (2.4) |  |  | 19 (1.1) |  |  | 32 (1.9) |  |  |
| C-reactive protein (mg/dL) | 0 (1704) | 37 (2.2) | 0.9 (0.5 – 1.9) | *0.8712* | 17 (1.0) | 0.9 (0.4 – 2.4) | *0.9069* | 22 (1.3) | 0.7 (0.4 – 1.2) | *0.1575* |
|  | 1 (1921) | 40 (2.1) |  |  | 21 (1.1) |  |  | 28 (1.5) |  |  |
| Cotinine (ng/mL) | 0 (1995) | 33 (1.7) | 1.4 (0.8 – 2.2) | *0.1925* | 15 (0.8) | 1.2 (0.6 – 2.6) | *0.5833* | 25 (1.3) | 0.9 (0.4 – 2.0) | *0.8464* |
|  | 1 (1621) | 44 (2.7) |  |  | 23 (1.4) |  |  | 25 (1.5) |  |  |
| Homocysteine (µmol/L) | 0 (1754) | 46 (2.6) | 1.2 (0.7 – 2.1) | *0.4481* | 24 (1.4) | 0.8 (0.4 – 1.8) | *0.5996* | 30 (1.7) | 1.0 (0.3 – 3.2) | *0.9758* |
|  | 1 (1885) | 32 (1.7) |  |  | 14 (0.7) |  |  | 19 (1.0) |  |  |
| Gamma glutamyl transferase (U/L) | 0 (1690) | 31 (1.8) | 1.3 (0.4 – 3.7) | *0.6268* | 12 (0.9 | 1.8 (0.6 – 5.1) | *0.2538* | 14 (0.8) | 2.5 (1.2 – 5.3) | *0.0200** |
|  | 1 (1909) | 46 (2.4) |  |  | 22 (1.2) |  |  | 35 (1.8) |  |  |
| Alkaline phosphatase (U/L) | 0 (1626) | 31 (1.9) | 1.1 (0.7 – 1.8) | *0.6270* | 16 (1.0) | 0.9 (0.5 – 1.9) | *0.8573* | 25 (1.5) | 0.5 (0.3 – 0.9) | *0.0252** |
|  | 1 (1974) | 46 (2.3) |  |  | 21 (1.1) |  |  | 24 (1.20 |  |  |
| Total calcium (mg/dL) | 0 (1533) | 39 (2.5) | 0.7 (0.4 – 1.4) | *0.3329* | 15 (1.0) | 1.8 (0.8 – 4.0) | *0.1333* | 17 (1.1) | 1.8 (0.4 – 2.0) | *0.2547* |
|  | 1 (2067) | 38 (1.8) |  |  | 22 (1.1) |  |  | 32 (1.6) |  |  |
| Bicarbonate (mmol/L) | 0 (1571) | 38 (2.4) | 0.9 (0.6 – 1.5) | *0.7285* | 18 (1.2) | 1.6 (0.9 – 2.9) | *0.1015* | 20 (1.3) | 2.0 (0.9 – 4.7) | *0.1012* |
|  | 1 (2029) | 39 (1.9) |  |  | 19 (0.9) |  |  | 29 (1.4) |  |  |
| Lactate dehydrogenase LDH (U/L) | 0 (1680) | 31 (1.9) | 1.6 (0.9 – 2.6) | *0.0754* | 18 (1.1) | 1.4 (0.6 – 3.4) | *0.4160* | 26 (1.6) | 1.0 (0.5 – 2.0) | *0.9937* |
|  | 1 (1915) | 46 (2.4) |  |  | 19 (1.0) |  |  | 23 (1.2) |  |  |
| Phosphorus (mg/dL) | 0 (1715) | 35 (2.0) | 1.4 (0.6 – 3.1) | *0.4090* | 20 (1.2) | 0.9 (0.5 – 1.9) | *0.8596* | 22 (1.3) | 1.4 (0.9 – 2.5) | *0.1526* |
|  | 1 (1884) | 42 (2.2) |  |  | 17 (0.9) |  |  | 27 (1.4) |  |  |
| Total protein (g/L) | 0 (1630) | 37 (2.3) | 1.2 (0.6 – 2.3) | *0.5315* | 19 (1.2) | 1.2 (0.6 – 2.4) | *0.6166* | 19 (1.2) | 1.6 (0.9 – 2.8) | *0.0888* |
|  | 1 (1967) | 40 (2.0) |  |  | 18 (0.9) |  |  | 30 (1.5) |  |  |
| Uric acid (mg/dL) | 0 (1754) | 42 (2.40 | 0.7 (0.4 – 1.3) | *0.2515* | 17 (1.0) | 1.2 (0.4 – 3.8) | *0.6779* | 23 (1.3) | 1.5 (0.7 – 3.5) | *0.2881* |
|  | 1 (1844) | 35 (1.9) |  |  | 20 (1.1) |  |  | 26 (1.4) |  |  |
| Sodium (mmol/L) | 0 (1363) | 39 (2.9) | 0.7 (0.3 – 1.4) | *0.2781* | 17 (1.3) | 0.8 (0.3 – 2.3) | *0.6578* | 18 (1.3) | 1.8 (0.5 – 5.9) | *0.3155* |
|  | 1 (2236) | 38 (1.7) |  |  | 20 (0.9) |  |  | 31 (1.4) |  |  |
| Potassium (mmol/L) | 0 (1654) | 38 (2.3) | 0.7 (0.3 – 1.4) | *0.2991* | 11 (0.7) | 1.6 (0.9 – 3.1) | *0.1280* | 22 (1.3) | 1.3 (0.6 – 2.6) | *0.5207* |
|  | 1 (1945) | 39 (2.0) |  |  | 26 (1.3) |  |  | 27 (1.4) |  |  |
| Chloride (mmol/L) | 0 (1599) | 34 (2.1) | 0.8 (0.4 – 1.3) | *0.2766* | 18 (1.1) | 0.5 (0.2 – 1.3) | *0.1684* | 20 (1.3) | 1.1 (0.6 – 2.0) | *0.7450* |
|  | 1 (2213) | 43 (2.2) |  |  | 19 (1.0) |  |  | 29 (1.5) |  |  |
| Globulin (g/dL) | 0 (1371) | 29 (2.1) | 1.2 (0.6 – 2.3) | *0.6166* | 12 (0.9) | 1.2 (0.6 – 2.5) | *0.3529* | 16 (1.2) | 1.1 (0.6 – 2.0) | *0.8441* |
|  | 1 (2221) | 48 (2.2) |  |  | 25 (1.1) |  |  | 33 (1.5) |  |  |

^a^ Participants with pain lasting ≥24 hours in the past month at one of the anatomical sites studied were asked for how long they experienced this pain: ≤1 month, between 1 and 3 months, at least 3 months but less than 1 year or ≥1 year. Participants who answered “≤1 month” were considered to have acute pain.

^b^ Total number of participants aged 20 years and older who were asked about musculoskeletal pain. Acute pain analyses were done on the population aged ≥20 years in our bank minus chronic and subacute pain ((4742 - (779+129)) = 3834.

^c^ All analyses are adjusted for sex (male, female), age (20–34; 35–49; 50–64; 65–79; ≥80 years), and BMI (<20; 20–24.9; 25–29.9; ≥30).

Biomarkers are dichotomized at the median of the distribution because when considered as continuous variables the validity of the model fit was often questionable.

^d^ Since NHANES data were weighted to make them comparable to those of the non-institutionalized US population, the proportions are not exactly 50% on each side of the median.
