# Supplementary material for: An exploratory identification of biological markers of chronic musculoskeletal pain in the low back, neck, and shoulders
Source: PLoS One. 2022 Apr 15;17(4):e0266999. doi: 10.1371/journal.pone.0266999 (PMC9012384; doi:10.1371/journal.pone.0266999)
Supplement: S2 Appendix — (DOCX) [file pone.0266999.s002.docx]

**APPENDIX 2:**

**Table 5. Results of multivariable analyses of the associations between biomarkers retained and the three subacute pain^a^ sites studied (n = 3,658^b^).**

| **Biomarker^c^** | **Class (n)^d^** | **Low back pain** | | | **Shoulder pain** | | | **Neck pain** | | |
| --- | --- | --- | --- | --- | --- | --- | --- | --- | --- | --- |
|  |  | **Frequency by class (%)** | **Odds Ratio (IC - 95%)** | ***P value*** | **Frequency by class (%)** | **Odds Ratio (IC - 95%)** | ***P value*** | **Frequency by class (%)** | **Odds Ratio (IC - 95%)** | ***P value*** |
| Acrylamide (pmoL/G Hb) | 0 (1683) | 13 (0.8) | 1.7 (0.6 – 4.9) | *0.3355* | 11 (0.7) | 1.4 (0.6 – 3.2) | *0.4799* | 9 (0.5) | 0.9 (0.3 – 2.1) | *0.7129* |
|  | 1 (1464) | 16 (1.1) |  |  | 14 (1.0) |  |  | 6 (0.4) |  |  |
| Glycidamide (pmoL/G Hb) | 0 (1709) | 13 (0.8) | 1.9 (0.7 – 5.2) | *0.1839* | 13 (0.8) | 1.1 (0.5 – 2.2) | *0.7937* | 11 (0.6) | 0.4 (0.1 – 1.6) | *0.1720* |
|  | 1 (1485) | 16 (1.1) |  |  | 13 (0.9) |  |  | 4 (0.3) |  |  |
| Albumin, urine (µg/mL) | 0 (1581) | 10 (0.6) | 1.8 (0.8 – 3.9) | *0.1400* | 9 (0.6) | 1.5 (0.6 – 3.7) | *0.3003* | 7 (0.4) | 1.3 (0.4 – 4.0) | *0.6337* |
|  | 1 (1967) | 23 (1.2) |  |  | 18 (0.9) |  |  | 10 (0.5) |  |  |
| Ascorbic acid (µmol/L) (Vitamin C) | 0 (1636) | 16 (1.0) | 0.7 (0.3 – 1.6) | *0.3582* | 15 (0.9) | 0.5 (0.2 – 1.5) | *0.2235* | 10 (0.6) | 0.7 (0.1 – 3.1) | *0.5810* |
|  | 1 (1783) | 14 (0.8) |  |  | 11 (0.6) |  |  | 7 (0.4) |  |  |
| Retinol (µg/dL) (Vitamin A) | 0 (1877) | 14 (0.8) | 1.4 (0.6 – 3.6) | *0.4323* | 11 (0.6) | 1.9 (0.7 – 5.4) | *0.2150* | 9 (0.5) | 0.9 (0.2 – 3.7) | *0.8362* |
|  | 1 (1550) | 15 (1.0) |  |  | 15 (1.0) |  |  | 7 (0.6) |  |  |
| α-Tocopherol (µg/dL)  (Vitamin E) | 0 (1674) | 13 (0.8) | 1.2 (0.6 – 2.4) | *0.8329* | 13 (0.8) | 0.8 (0.3 – 2.1) | *0.6830* | 7 (0.4) | 1.1 (0.3 – 4.4) | *0.9119* |
|  | 1 (1753) | 16 (0.9) |  |  | 13 (0.7) |  |  | 9 (5) |  |  |
| α-Carotene (µg/dL) | 0  (1571) | 12 (0.8) | 0.8 (0.3 – 2.1) | *0.5816* | 16 (1.0) | 0.3 (0.1 – 1.0) | *0.0501* | 10 (0.6) | 0.3 (0.1 – 1.9) | *0.2109* |
|  | 1  (1855) | 17 (0.9) |  |  | 10 (0.5) |  |  | 6 (0.3) |  |  |
| β-Carotene (µg/dL) | 0 (1564) | 13 (0.8) | 0.9 (0.4 – 2.4) | *0.8472* | 19 (1.2) | 0.2 (0.1 – 0.4) | *0.009** | 10 (0.6) | 0.5 (0.1 – 2.3) | *0.3298* |
|  | 1 (1863) | 16 (0.9) |  |  | 7 (0.4) |  |  | 6 (0.3) |  |  |
| Cadmium (µg/L) | 0 (1636) | 13 (0.8) | 1.3 (0.4 – 4.8) | *0.6761* | 12 (0.7) | 0.9 (0.4 – 2.0) | *0.7671* | 8 (0.5) | 0.7 (0.2 – 2.6) | *0.5481* |
|  | 1 (1841) | 17 (0.9) |  |  | 15 (0.8) |  |  | 9 (0.5) |  |  |
| Lead (µg/dL) | 0 (1482) | 14 (0.9) | 0.7 (0.3 – 1.5) | *0.2883* | 8 (0.5) | 1.2 (0.3 – 4.5) | *0.7741* | 5 (0.3) | 1.9 (0.7 – 5.4) | *0.2135* |
|  | 1 (1995) | 16 (0.8) |  |  | 19 (1.0) |  |  | 12 (0.6) |  |  |
| Mercury, total (µg/L) | 0 (1757) | 15 (0.9) | 0.8 (0.3 – 2.1) | *0.5875* | 19 (1.1) | 0.3 (0.1 – 0.8) | *0.0176** | 12 (0.7) | 0.6 (0.1 – 2.4) | *0.4393* |
|  | 1 (1720) | 15 (0.9) |  |  | 8 (0.5) |  |  | 5 (0.3) |  |  |
| Triglycerides (mg/dL) | 0 (1683) | 14 (0.8) | 1.1 (0.4 – 3.1) | *0.8480* | 13 (0.8) | 1.3 (0.4 – 4.0) | *0.6251* | 9 (0.5) | 0.5 (0.2 – 1.6) | *0.2607* |
|  | 1 (1740) | 14 (0.8) |  |  | 12 (0.7) |  |  | 7 (0.4) |  |  |
| Direct HDL-cholesterol (mg/dL) | 0 (1632) | 13 (0.8) | 0.7 (0.2 – 1.8) | *0.3820* | 10 (0.6) | 1.6 (0.9 – 2.8) | *0.1177* | 6 (0.4) | 1.2 (0.2 – 9.4) | *0.8232* |
|  | 1 (1812) | 17 (0.9) |  |  | 16 (0.9) |  |  | 11 (0.6) |  |  |
| Total Cholesterol (mg/dL) | 0 (1682) | 15 (0.9) | 1.1 (0.5 – 2.6) | *0.8052* | 8 (0.5) | 3.1 (0.9 – 10.9) | *0.8857* | 6 (0.4) | 3.1 (1.1 – 8.4) | *0.0322** |
|  | 1 (1763) | 15 (0.9) |  |  | 18 (1.0) |  |  | 11 (0.6) |  |  |
| White blood cell count (1000 cells/µL) | 0 (1754) | 9 (0.5) | 2.4 (0.9 – 6.3) | *0.0698* | 14 (0.8) | 1.2 (0.6 – 2.5) | *0.6384* | 8 (0.5) | 1.2 (0.2 – 7.1) | *0.8117* |
|  | 1 (1727) | 21 (1.2) |  |  | 13 (0.8) |  |  | 9 (0.5) |  |  |
| Platelet count SI (1000 cells/µL) | 0 (1861) | 14 (0.8) | 1.3 (0.5 – 3.1) | *0.5373* | 17 (0.9) | 0.8 (0.2 – 3.7) | *0.7780* | 7 (0.4) | 1.5 (0.7 – 3.0) | *0.3789* |
|  | 1 (1620) | 16 (1.0) |  |  | 10 (0.6) |  |  | 10 (0.6) |  |  |
| C-reactive protein (mg/dL) | 0 (1614) | 12 (0.7) | 1.8 (0.5 – 6.4) | *0.3687* | 11 (0.7) | 2.2 (0.8 – 6.4) | *0.1252* | 10 (0.6) | 0.7 (0.3 – 1.9) | *0.4875* |
|  | 1 (1838) | 17 (0.9) |  |  | 15 (0.8) |  |  | 7 (0.4) |  |  |
| Cotinine (ng/mL) | 0 (1904) | 15 (0.8) | 2.0 (0.6 – 6.2) | *0.2205* | 12 (0.6) | 1.6 (0.7 – 3.7) | *0.2233* | 8 (0.4) | 1.1 (0.2 – 7.8) | *0.8829* |
|  | 1 (1541) | 15 (1.0) |  |  | 14 (0.9) |  |  | 9 (0.6) |  |  |
| Homocysteine (µmol/L) | 0 (1640) | 15 (0.9) | 0.9 (0.3 – 2.8) | *0.8639* | 11 (0.7) | 1.5 (0.7 – 3.3) | *0.3054* | 9 (0.6) | 0.9 (0.2 – 4.1) | *0.8855* |
|  | 1 (1827) | 15 (0.8) |  |  | 16 (0.9) |  |  | 8 (0.4) |  |  |
| Gamma glutamyl transferase (U/L) | 0 (1605) | 10 (0.6) | 1.9 (0.7 – 4.9) | *0.1630* | 12 (0.8) | 1.4 (0.5 – 3.5) | *0.5034* | 4 (0.3) | 4.1 (1.2 – 13.8) | *0.0241** |
|  | 1 (1820) | 18 (1.0) |  |  | 13 (0.7) |  |  | 12 (0.7) |  |  |
| Alkaline phosphatase (U/L) | 0 (1547) | 8 (0.5) | 2.0 (0.7 – 6.0) | *0.1844* | 7 (0.5) | 1.8 (0.9 – 3.7) | *0.0746* | 4 (0.3) | 1.5 (0.2 – 10.5) | *0.6541* |
|  | 1 (1879) | 20 (1.1) |  |  | 18 (1.0) |  |  | 12 (0.6) |  |  |
| Total calcium (mg/dL) | 0 (1445) | 15 (1.0) | 0.7 (0.3 – 1.9) | *0.5237* | 12 (0.8) | 0.7 (0.2 – 1.9) | *0.4129* | 6 (0.4) | 1.0 (0.2 – 3.8) | *0.9665* |
|  | 1 (1981) | 13 (07) |  |  | 13 (0.7) |  |  | 10 (0.5) |  |  |
| Bicarbonate (mmol/L) | 0 (1494) | 17 (1.1) | 0.3 (0.1 – 0.8) | *0.0141** | 9 (0.6) | 1.1 (0.4 – 2.9) | *0.8910* | 9 (0.6) | 0.4 (0.2 – 1.2) | *0.0944* |
|  | 1 (1932) | 11 (0.6) |  |  | 16 (0.8) |  |  | 7 (0.4) |  |  |
| Lactate dehydrogenase LDH (U/L) | 0 (1589) | 11 (0.7) | 1.4 (0.6 – 3.2) | *0.4541* | 9 (0.6) | 1.6 (0.6 – 4.4) | *0.3618* | 6 (0.4) | 1.5 (0.3 – 7.0) | *0.6005* |
|  | 1 (1833) | 17 (0.9) |  |  | 16 (0.9) |  |  | 10 (0.6) |  |  |
| Phosphorus (mg/dL) | 0 (1636) | 16 (1.0) | 0.7 (0.3 – 1.4) | *0.2404* | 12 (0.7) | 1.2 (0.5 – 2.7) | *0.6712* | 7 (0.4) | 1.4 (0.6 – 3.4) | *0.4226* |
|  | 1 (1789) | 12 (0.7) |  |  | 13 (0.7) |  |  | 9 (0.5) |  |  |
| Total protein (g/L) | 0 (1535) | 11 (0.7) | 1.3 (0.4 – 4.4) | *0.7020* | 10 (0.6) | 0.8 (0.3 – 2.2) | *0.7183* | 5 (0.3) | 2.0 (0.6 – 6.4) | *0.2064* |
|  | 1 (1889) | 17 (0.9) |  |  | 15 (0.8) |  |  | 11 (0.6) |  |  |
| Uric acid (mg/dL) | 0 (1665) | 19 (1.1) | 0.4 (0.2 – 1.2) | *0.0946* | 16 (1.0) | 0.9 (0.4 – 2.3) | *0.8032* | 11 (0.7) | 0.2 (0.06 – 1.4) | *0.1144* |
|  | 1 (1759) | 9 (0.5) |  |  | 9 (0.5) |  |  | 5 (0.3) |  |  |
| Sodium (mmol/L) | 0 (1293) | 14 (1.1) | 0.6 (0.2 – 2.1) | *0.4212* | 9 (0.7) | 0.6 (0.3 – 1.4) | *0.2437* | 6 (0.5) | 0.7 (0.1 – 3.0) | *0.5617* |
|  | 1 (2133) | 14 (0.7) |  |  | 16 (0.8) |  |  | 10 (0.5) |  |  |
| Potassium (mmol/L) | 0 (1561) | 13 (0.8) | 1.4 (0.6 – 3.4) | *0.3959* | 13 (0.8) | 1.2 (0.4 – 3.7) | *0.6887* | 6 (0.4) | 2.1 (0.5 – 9.6) | *0.2947* |
|  | 1 (1864) | 15 (0.8) |  |  | 12 (0.6) |  |  | 10 (0.5) |  |  |
| Chloride (mmol/L) | 0 (1524) | 13 (0.9) | 1.2 (0.4 – 3.6) | *0.7345* | 13 (0.9) | 0.8 (0.3 – 2.3) | *0.5979* | 6 (0.4) | 0.9 (0.3 – 3.3) | *0.8971* |
|  | 1 (1902) | 15 (0.8) |  |  | 12 (0.6) |  |  | 10 (0.5) |  |  |
| Globulin (g/dL) | 0 (1313) | 12 (0.9) | 0.9 (0.4 – 2.1) | *0.7449* | 10 (0.8) | 1.1 (0.4 – 3.3) | *0.8865* | 5 (0.4) | 0.9 (0.3 – 2.7) | *0.8662* |
|  | 1 (2111) | 16 (0.8) |  |  | 15 (0.7) |  |  | 11 (0.5) |  |  |

^a^ Participants with pain lasting ≥24 hours in the past month at one of the anatomic sites studied were asked for how long they experienced this pain: ≤1 month, between 1 and 3 months, at least 3 months but less than 1 year or ≥1 year. Participants who answered “between 1 and 3 months” were considered to have subacute pain.

^b^ Total number of patients aged ≥20 years who were asked about musculoskeletal pain. Subacute pain analyses were done on the population aged ≥20 years in our bank minus chronic and acute pain ((4742 - (779+305)) = 3658. Since there were missing data on some biomarkers, class (n) column presents the sample size on which the analyses for each biomarker were done.

^c^ All analyses adjusted for sex (male, female), age (20–34; 35–49; 50–64; 65–79; ≥80 years), and BMI (<20; 20–24.9; 25–29.9; ≥30). Biomarkers are dichotomized at the median of the distribution because when considered as continuous variables the validity of the model fit was often questionable.

^d^ Since NHANES data were weighted to make them comparable to those of the non-institutionalized US population, the proportions are not exactly 50% on each side of the median.
