# Supplementary material for: An exploratory identification of biological markers of chronic musculoskeletal pain in the low back, neck, and shoulders
Source: PLoS One. 2022 Apr 15;17(4):e0266999. doi: 10.1371/journal.pone.0266999 (PMC9012384; doi:10.1371/journal.pone.0266999)
Supplement: S3 Appendix — (DOCX) [file pone.0266999.s003.docx]

**APPENDIX 3**: **List of specific biomarkers considered in NHANES 2003–2004.**

1. Vitamins and urinary markers

| **Variables** | **Labels** |
| --- | --- |
| LBXACR | Acrylamide (pmoL/G Hb) |
| LBXGLY | Glycidamide (pmoL/G Hb) |
| URXUCR | Creatinine, urine (mg/dL) |
| URXUMA | Albumin, urine (ug/mL) |
| URXUAS | Urinary total arsenic (µg/L) |
| URXUAS3 | Urinary arsenous acid (µg/L) |
| URXUAS5 | Urinary arsenic acid (µg/L) |
| URXUAB | Urinary arsenobetaine (µg/L) |
| URXUAC | Urinary arsenocholine (µg/L) |
| URXUDMA | Urinary dimethylarsonic acid (µg/L) |
| URXUMMA | Urinary monomethylacrsonic acid |
| URXUTM | Urinary trimethylarsine Oxide (µg/L) |
| LBDVICSI | Ascorbic acid (umol/L) (Vitamin C) |
| LBXVIA | Retinol(ug/dL) (Vitamin A) |
| LBXATC | a-Tocopherol (ug/dL) (Vitamin E) |
| LBXALC | a-Carotene (ug/dL) |
| LBXBCC | β-Carotene (ug/dL) |
| LBXVB6 | Vitamin B6 (nmol/L) |
| LBDVIDMS | Vitamin D (nmol/L) |

1. Brominated flame retardants (BFRs)

| **Variables** | **Labels** |
| --- | --- |
| LBXBB1 | 2,2’,4,4’,5,5’-hexabromobiphenyl |
| LBXBB1LA | 2,2’,4,4’,5,5’-hexbrombiphenyl lipid adj |
| LBXBR1 | 2,2’,4-tribromodiphenyl ether |
| LBXBR1LA | 2,2’,4-tribromodiphenyl ether lipid adj |
| LBXBR2 | 2,4,4’-tribromodiphenyl ether |
| LBXBR2LA | 2,4,4’-tribromodiphenyl ether lipid adj |
| LBXBR3 | 2,2’,4,4’-tetrabromodiphenyl ether |
| LBXBR3LA | 2,2’,4,4’-tetrabromphenyl ether lipid ad |
| LBXBR4 | 2,2’,3,4,4’-pentabromodiphenyl ether |
| LBXBR4LA | 2,2’,3,4,4’-pentabromphenyl lipid adj |
| LBXBR5 | 2,2’,4,4’,5-pentabromodiphenyl ether |
| LBXBR5LA | 2,2’,4,4’,5-pentabromphenyl lipid adj |
| LBXBR6 | 2,2’,4,4’,6-pentabromodiphenyl ether |
| LBXBR6LA | 2,2’,4,4’,6-pentabromdphenyl lipid adj |
| LBXBR7 | 2,2’,4,4’,5,5’-hexabromodiphenyl ether |
| LBXBR7LA | 2,2’,4,4’,5,5’-hexabromphenyl lipid adj |
| LBXBR8 | 2,2’,4,4’,5,6’-hexabromodiphenyl ether |
| LBXBR8LA | 2,2’,4,4’,5,6’-hexabromphenyl lipid adj |
| LBXBR9 | 2,2’,3,4,4’,5’,6-heptabromodiphenyl ethr |
| LBXBR9LA | 2,2’,3,4,4’,5’,6-heptabrophenl lipid adj |
| LBXBR66 | 2,3’,4,4’-tetrabromodiphenyl ether |
| LBXBR66L | 2,3’,4,4’-tetrabromodiphenyl lipid adj |

1. Cadmium, lead and total mercury (blood test)

| **Variables** | **Labels** |
| --- | --- |
| LBXBCD | Cadmium (ug/L) |
| LBXBPB | Lead (ug/dL) |
| LBXTHG | Mercury, total (ug/L) |
| LBXIHG | Mercury, inorganic (ug/L) |

1. Cholesterol - total

| **Variables** | **Labels** |
| --- | --- |
| LBXTC | Total cholesterol(mg/dL) |
| LBXHDD | Direct HDL-cholesterol (mg/dL) |

1. Complete blood count with 5-part differential - whole blood (L25_C)

| **Variables** | **Labels** |
| --- | --- |
| LBXWBCSI | White blood cell count (1000 cells/uL) |
| LBXRBCSI | Red blood cell count (million cells/uL) |
| LBXHGB | Hemoglobin (g/dL) |
| LBXPLTSI | Platelet count SI (1000 cells/uL) |

1. C - reactive protein (CRP) and parathyroid hormone (PTH) (markers of inflammation)

| **Variables** | **Labels** |
| --- | --- |
| LBXCRP | C-reactive protein (mg/dL) |
| LBXPT21 | Parathyroid hormone (Elecys method) pg/mL |

1. Cotinine

| **Variables** | **Labels** |
| --- | --- |
| LBXCOT | Cotinine (ng/mL) |

1. Environmental phenols (L24EPH_C)

| **Variables** | **Labels** |
| --- | --- |
| URXBPH | Urinary bisphenol A (ng/mL) |
| URXBP3 | Urinary benzophenone-3 (ng/mL) |
| URDTRS | Urinary triclosan (ng/mL) |

1. Selenium

| **Variables** | **Labels** |
| --- | --- |
| LBXSEL | Selenium (ug/L) |
| LBDSELSI | Selenium (umol/L) |

1. Glycohemoglobin (%) and folate - RBC serum and vitamin B12 (vitamins)

| **Variables** | **Labels** |
| --- | --- |
| LBXRBF | Folate, RBC (ng/mL RBC) |
| LBXGH | Glycohemoglobin (%) |
| **LBXFOL** | Folate, serum (ng/mL) |

1. Iodine - urine (L06UIO_C) and mercury - inorganic, urine (L06UHG_C)

| **Variables** | **Labels** |
| --- | --- |
| **URXUIO** | Iodine, urine (ng/mL) |
| **URXUHG** | Mercury, urine (ng/mL) |

1. Metals - urine

| **Variables** | **Labels** |
| --- | --- |
| URXUBA | Barium, urine (ng/mL) |
| URXUBE | Beryllium, urine (ng/mL) |
| URXUCD | Cadmium, urine (ng/mL) |
| URXUCO | Cobalt, urine (ng/mL) |
| URXUCS | Cesium, urine (ng/mL) |
| URXUMO | Molybdenum, urine (ng/mL) |
| URXUPB | Lead, urine (ng/mL) |
| URXUPT | Platinum, urine (ng/mL) |
| URXUSB | Antimony, urine (ng/mL) |
| URXUTU | Tungsten, urine (ng/mL) |
| URXUUR | Uranium, urine (ng/mL) |

1. Methyl malonic acid and homocysteine

| **Variables** | **Labels** |
| --- | --- |
| LBXHCY | Homocysteine (umol/L) |
| LBXMMA | Methylmalonic acid (umol/L) |

1. Polychlorinated biphenyls not related to dioxins

| **Variables** | **Labels** |
| --- | --- |
| LBX044LA | PCB44 Lipid Adj (ng/g) |
| LBX049LA | PCB49 Lipid Adj (ng/g) |
| LBX052LA | PCB52 Lipid Adj (ng/g) |
| LBX087LA | PCB87 Lipid Adj (ng/g) |
| LBX099LA | PCB99 Lipid Adj (ng/g) |
| LBX101LA | PCB101 Lipid Adj (ng/g) |
| LBX110LA | PCB110 Lipid Adj (ng/g) |
| LBX128LA | PCB128 Lipid Adj (ng/g) |
| LBX138LA | PCB138 & 158 Lipid Adj (ng/g) |
| LBX146LA | PCB146 Lipid Adj (ng/g) |
| LBX149LA | PCB149 Lipid Adj (ng/g) |
| LBX151LA | PCB151 Lipid Adj (ng/g) |
| LBX153LA | PCB153 Lipid Adj (ng/g) |
| LBX170LA | PCB170 Lipid Adj (ng/g) |
| LBX172LA | PCB172 Lipid Adj (ng/g) |
| LBX177LA | PCB177 Lipid Adj (ng/g) |
| LBX178LA | PCB178 Lipid Adj (ng/g) |
| LBX180LA | PCB180 Lipid Adj (ng/g) |
| LBX183LA | PCB183 Lipid Adj (ng/g) |
| LBX187LA | PCB187 Lipid Adj (ng/g) |
| LBX194LA | PCB194 Lipid Adj (ng/g) |
| LBX195LA | PCB195 Lipid Adj (ng/g) |
| LBX196LA | PCB196 & 203 Lipid Adj (ng/g) |
| LBD199LA | PCB199 Lipid Adj (ng/g) |
| LBX206LA | PCB206 Lipid Adj (ng/g) |
| LBX209LA | PCB209 Lipid Adj (ng/g) |

1. Organophosphorus insecticides, perchlorate

| **Variables** | **Labels** |
| --- | --- |
| URXOP1 | Dimethylphosphate (µg/L) |
| URXOP2 | Diethylphosphate (µg/L) |
| URXOP3 | Dimethylthiophosphate (µg/L) |
| URXOP4 | Diethylthiophosphate (µg/L) |
| URXOP5 | Dimethyldithiophosphate (µg/L) |
| URXOP6 | Diethyldithiophosphate (µg/L) |
| URXUCR | Creatinine, urine (mg/dL) |
| URXUP8 | Perchlorate, urine (ng/mL) |

1. Pesticides

| **Variables** | **Labels** |
| --- | --- |
| URX24D | 2,4-D (ug/L) |
| URX25T | 2,4,5 Trichlorophenoxyacetic acid (ug/L) |
| URXCBF | Carbofuranphenol (ug/L) |
| URXEMM | Ethametsulfuron methyl (ug/L) |
| URXETU | Ethylenethio urea (ug/L) |
| URXMMI | Methamidaphos (ug/L) |
| URXMTM | Metsulfuron methyl (ug/L) |
| URXMTO | Dimethoate (ug/L) |
| URXPCP | Pentachlorophenol (ug/L) |
| URXPRO | Prosulfuron (ug/L) |
| URXPTU | Propylenethio urea (ug/L) |
| URXSSF | Sulfosulfuron (ug/L) |

1. Pesticides - environmental - urine (L24PP_C)

| **Variables** | **Labels** |
| --- | --- |
| URX14D | 2,5-dichlorophenol (ug/L) result |
| URXOPP | O-Phenyl phenol (ug/L) result |
| URXDCB | 2,4-dichlorophenol (ug/L) result |
| URX1TB | 2,4,5-trichlorophenol (ug/L) result |
| URX3TB | 2,4,6-trichlorophenol (ug/L) result |

1. Pesticides - organochlorine metabolites - serum (excess) (L28OCP_C)

| **Variables** | **Labels** |
| --- | --- |
| LBXHCBLA | Hexachlorobenzene Lipid Adj |
| LBXBHCLA | B-hexachlorocyclohexane Lipid Adj (ng/g) |
| LBXGHCLA | G-hexachlorocyclohexane Lipid Adj (ng/g) |
| LBXPDELA | p,p’-DDE Lipid Adj (ng/g) |
| LBXPDTLA | p,p’-DDT Lipid Adj (ng/g) |
| LBXODTLA | o,p’-DDT Lipid Adj (ng/g) |
| LBXTNALA | Trans-nonachlor Lipid Adj (ng/g) |
| LBXHPELA | Heptachlor Epoxide Lipid Adj (ng/g) |
| LBXMIRLA | Mirex Lipid Adj (ng/g) |
| LBXALDLA | Aldrin Lipid Adj (ng/g) |
| LBXDIELA | Dieldrin Lipid Adj (ng/g) |
| LBXENDLA | Endrin Lipid Adj (ng/g) |

1. Phtalates

| **Variables** | **Labels** |
| --- | --- |
| URXMBP | Mono-n-butyl phthalate |
| URXMEP | Mono-ethyl phthalate |
| URXMHP | Mono-(2-ethyl)-hexyl phthalate |
| URXECP | Mono-2-ethyl-5-carboxypentyl phthalate |

1. Phytoestrogens - urine

| **Variables** | **Label** |
| --- | --- |
| URXDAZ | Daidzein (ng/mL) |
| URXDMA | o-Desmethylangolensin (O-DMA) (ng/mL) |
| URXEQU | Equol (ng/mL) |
| URXETD | Enterodiol (ng/mL) |
| URXETL | Enterolactone (ng/mL) |
| URXGNS | Genistein (ng/mL) |

1. Fasting glucose, serum peptide, insulin (L10AM_C)

| **Variables** | **Labels** |
| --- | --- |
| LBXGLU | Glucose, plasma (mg/dL) |
| LBXCPSI | C-peptide: SI (nmol/L) |
| LBXIN | Insulin (uU/mL) |

1. Polyfluoroalkyl chemicals (L24PFC_C)

| **Variables** | **Labels** |
| --- | --- |
| LBXPFOA | Perfluorooctanoic acid |
| LBXPFOS | Perfluorooctane sulfonic acid |
| LBXPFHS | Perfluorohexane sulfonic acid |
| LBXEPAH | 2-(N-ethyl-PFOSA) acetate |
| LBXMPAH | 2-(N-methyl-PFOSA) acetate |
| LBXPFDE | Perfluorodecanoic acid |
| LBXPFBS | Perfluorobutane sulfonic acid |
| LBXPFHP | Perfluoroheptanoic acid |
| LBXPFNA | [Perfluorononanoic acid](https://wwwn.cdc.gov/Nchs/Nhanes/2003-2004/L24PFC_C.htm#LBXPFNA) |
| LBXPFSA | Perfluorooctane sulfonamide |
| LBXPFUA | Perfluoroundecanoic acid |
| LBXPFDO | Perfluorododecanoic acid |

1. Standard biochemicals (L40_C)

| **Variables** | **Labels** |
| --- | --- |
| LBXSAL | Albumin (g/dL) |
| LBXSATSI | Alanine aminotransferase ALT (U/L) |
| LBXSASSI | Aspartate aminotransferase AST (U/L) |
| LBXSAPSI | Alkaline phosphatase (U/L) |
| LBXSBU | Blood urea nitrogen (mg/dL) |
| LBXSCA | Total calcium (mg/dL) |
| LBXSCH | Cholesterol (mg/dL) |
| LBXSC3SI | Bicarbonate (mmol/L) |
| LBXSGTSI | Gamma glutamyl transferase (U/L) |
| LBXSGL | Glucose, serum (mg/dL) |
| LBXSIR | Iron, refrigerated (ug/dL) |
| LBXSLDSI | Lactate dehydrogenase LDH (U/L) |
| LBXSPH | Phosphorus (mg/dL) |
| LBXSTB | Total bilirubin (mg/dL) |
| LBXSTP | Total protein (g/L) |
| LBXSTR | Triglycerides (mg/dL) |
| LBXSUA | Uric acid (mg/dL) |
| LBXSCR | Creatinine (mg/dL) |
| LBXSNASI | Sodium (mmol/L) |
| LBXSKSI | Potassium (mmol/L) |
| LBXSCLSI | Chloride (mmol/L) |
| LBXSOSSI | Osmolality (mmol/Kg) |
| LBXSGB | Globulin (g/dL) |
